# Artificial Intelligence–Based Echocardiographic Assessment of Diastolic Dysfunction: A Systematic Review

**DOI:** 10.1111/echo.70630

**Published:** 2026-09-23

**Authors:** Cian P. Murray, Hugo C. Temperley, Rob S. Doyle, Abdullahi Khair, Michal Jagiello, Amal John, Patrick O'Callaghan

**Affiliations:** ^1^ Cardiology Department Waterford University Hospital Waterford Leinster Ireland; ^2^ Trinity College Dublin Trinity Translational Medicine Institute Dublin Leinster Ireland; ^3^ Cardiology Department Mater Misericordiae University Hospital Dublin Leinster Ireland

**Keywords:** artificial intelligence, diagnostic accuracy, diastolic dysfunction, echocardiography, heart failure with preserved ejection fraction

## Abstract

Accurate non‐invasive assessment of left ventricular diastolic dysfunction and filling pressures is central to the diagnosis and management of heart failure, particularly heart failure with preserved ejection fraction (HFpEF). Despite widespread use of transthoracic echocardiography, guideline‐based algorithms are frequently indeterminate, variably reproducible, and difficult to apply consistently in routine practice. Artificial intelligence (AI) has emerged as a potential strategy to standardize measurement, integrate multiparametric echocardiographic data, and reduce diagnostic uncertainty. We performed a systematic review of diagnostic accuracy studies evaluating AI or machine‐learning approaches applied to echocardiography for automated assessment of diastolic function or estimation of left ventricular filling pressures. Databases were searched from inception to July 7, 2026. Reference standards included invasive hemodynamics or guideline‐based diastolic classification. Risk of bias and applicability were assessed using QUADAS‐3. Fourteen studies were included, with development samples and validation or test cohorts reported separately. Validation ranged from small held‐out invasive cohorts to large independent external datasets. Against invasive hemodynamic reference standards, reported AUCs ranged from 0.761 to 0.883, reflecting heterogeneous hemodynamic targets, thresholds, and validation designs. Studies evaluating agreement with guideline‐ or expert‐derived classifications and automated replication of individual diastolic parameters generally reported high discrimination, although these represented distinct clinical tasks and varied substantially in the independence and scale of validation. Several studies demonstrated external or prospective validation, but robust evaluation against invasive hemodynamics remained limited. Given substantial methodological and clinical heterogeneity, findings were synthesized descriptively and no summary estimate was calculated. Despite promising diagnostic discrimination in retrospective studies, AI‐based echocardiographic diastolic assessment remains limited by heterogeneous reference standards, variable validation approaches, and an absence of prospective interventional clinical‐impact studies and limited prospective multicenter validation against invasive hemodynamics, precluding routine implementation at this stage.

## Graphical abstract

AI‐based echocardiographic assessment of diastolic dysfunction demonstrates moderate‐to‐high diagnostic discrimination against invasive and guideline‐based reference standards and consistently resolves indeterminate classifications. Prospective multicenter validation, calibration reporting, and clinical impact data are required before routine adoption.

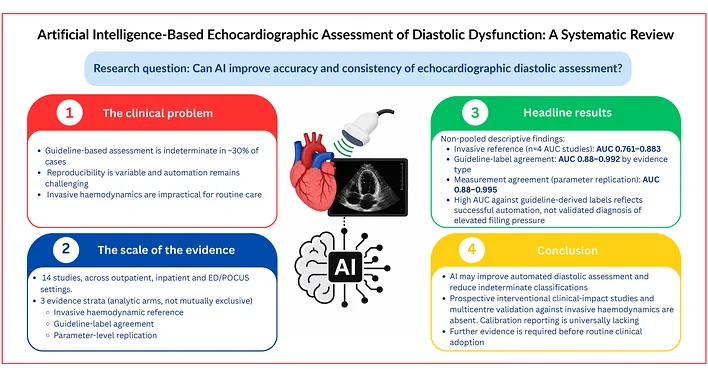

## 1 Introduction

Left ventricular diastolic dysfunction is a key pathophysiologic mechanism underlying dyspnea, exercise intolerance, and heart failure, especially in patients with preserved ejection fraction (HFpEF) [1, 2]. HFpEF comprises roughly half of all heart‐failure cases and, particularly in older adults, is associated with substantial morbidity and mortality [3]. In HFpEF, impaired relaxation and reduced ventricular compliance lead to elevated left‐ventricular filling pressures that drive symptoms, hospitalization, and adverse outcomes. Precise identification of raised filling pressures is therefore essential for diagnosis, risk stratification, and therapeutic decision‐making [2, 4]. Transthoracic echocardiography (TTE) is routinely used to estimate filling pressures, but non‐invasive assessment is limited by image quality, rhythm dependence, and missing or discordant parameters, often yielding indeterminate classifications [5]. This contributes to delayed or missed diagnoses and heterogeneous decision‐making [2, 3].

Current recommendations advocate a multiparametric algorithm integrating transmitral inflow (E/A ratio), tissue Doppler (e′ velocity, E/e′ ratio), left atrial volume or strain, and tricuspid regurgitation velocity. However, these measurements are variably affected by technical and patient factors, and up to 30% of cases remain indeterminate using these criteria, while inter‐observer variability further reduces reproducibility and confidence in guideline grading [3, 4, 6, 7]. Moreover, current guideline algorithms were developed for expert manual interpretation rather than automated or probabilistic implementation, contributing to variability and indeterminate outputs when applied at scale [8, 9]. When non‐invasive assessment is inconclusive, invasive hemodynamic assessment, including PCWP by right‐heart catheterization or LVEDP by left‐heart catheterization, remains the reference standard, although thresholds and clinical interpretation vary. Invasive assessment is impractical for routine use due to cost, availability, and procedural risks [1, 3].

In this context, artificial intelligence (AI)–based approaches have pursued two complementary objectives: automated replication of guideline‐based diastolic assessment and direct estimation of left‐ventricular filling pressure using invasive or surrogate reference standards. AI has the potential to advance diastolic assessment through both automated quantification and integrated, model‐based interpretation. Computer‐vision algorithms can standardize view selection and automatically extract Doppler and strain indices, reducing inter‐operator variability and workload [10, 11, 12]. Reflecting this, the American Society of Echocardiography (ASE) has acknowledged the role of AI in streamlining diastolic assessment [8]. Supervised or semi‐supervised models can then fuse heterogeneous signals, such as 2‐D cine, spectral Doppler, and strain into probabilistic estimates of diastolic grade or the likelihood of elevated left‐ventricular filling pressure. Early studies suggest fewer indeterminate outputs and improved reproducibility, but heterogeneity in targets, inputs, reference standards, and validation strategies limits synthesis and clinical translation [13, 14, 15, 16, 17, 18, 19, 20, 21, 22, 23, 24, 25, 26].

Recent primary studies have demonstrated the feasibility of fully automated AI‐enabled echocardiographic interpretation across multiple tasks [10, 11], and prior narrative reviews have examined AI applications in echocardiography broadly [12], but no systematic review has focused specifically on AI‐based diastolic function assessment and filling pressure estimation with formal risk‐of‐bias appraisal. Accordingly, we conducted a systematic review of AI tools that automate echocardiographic assessment of diastolic function and estimate left‐ventricular filling pressures, applying QUADAS‐3 across a focused, prespecified evidence base of diagnostic accuracy studies [27]. We synthesized diagnostic performance stratified by reference standard and clinical context, compared AI outputs with clinician assessment, and appraised risk of bias and applicability. Our aim was to guide future research by highlighting methodological limitations, sources of heterogeneity, and areas for improvement to support the eventual implementation of this technology in clinical practice.

## 2 Methods

### 2.1 Protocol and Registration

This systematic review was designed and reported in accordance with the Preferred Reporting Items for Systematic Reviews and Meta‐Analyses extension for Diagnostic Test Accuracy studies (PRISMA‐DTA) (supplementary material S7). The protocol was prospectively registered with PROSPERO (registration number: CRD420251140771).

### 2.2 Eligibility Criteria

Eligibility criteria were prespecified using a PICO framework. The index test and reference standard are specified as distinct concepts below, consistent with PRISMA‐DTA reporting requirements.

1. Population
  Adults (≥18 years) undergoing TTE in any clinical setting, including outpatient echocardiography laboratories, inpatient wards, and point‐of‐care ultrasound (POCUS) environments.
2. Index test
  Artificial intelligence or machine‐learning (AI/ML) systems operating on echocardiographic data—raw image or video data, spectral or tissue Doppler, derived strain measures, or tabular echocardiographic variables—to either:
  - automate measurement or grading of left ventricular diastolic function (LVDF), and/or
  - estimate left ventricular filling pressure (LVFP).
3. Target condition
  The target conditions addressed by this review are:
  - Elevated left ventricular filling pressure;
  - Left ventricular diastolic dysfunction of any grade;
  - Individual diastolic parameter values at prespecified clinical thresholds.
4. Reference standard
  The reference standards accepted for inclusion were:
  - Invasive hemodynamic measurements, specifically pulmonary capillary wedge pressure (PCWP) obtained by right‐heart catheterization, or left ventricular end‐diastolic pressure (LVEDP) obtained by left‐heart catheterization, at rest or during exercise, using prespecified thresholds; or
  - Guideline‐based LVDF grading or indices derived using ASE or ASE/European Association of Cardiovascular Imaging (EACVI) 2016 recommendations; [8] or
  - Expert manual echocardiographic measurements used as reference standards for automated parameter extraction or measurement replication studies.
5. Comparator
  Where applicable, clinician‐derived echocardiographic measurements, expert assessments, or outputs of guideline‐based algorithms.
6. Outcomes
  The primary outcome was diagnostic discrimination, quantified as the area under the receiver operating characteristic curve (AUC), for:
  - elevated LVFP compared with an invasive reference standard, and/or
  - detection or grading of LV diastolic dysfunction compared with guideline‐based reference standards.
7. Study design
  Prospective or retrospective diagnostic accuracy studies conducted in single‐ or multi‐center settings, including prospective observational emergency department or POCUS evaluations. We excluded case reports, narrative reviews, editorials, methodological studies without clinical testing, preprints, and abstract‐only conference reports.

### 2.3 Search Strategy

We systematically searched MEDLINE (via PubMed), Embase, Web of Science Core Collection, and IEEE Xplore from database inception to July 7, 2026. Search strategies combined controlled vocabulary and free‐text terms relating to echocardiography, diastolic function, filling pressures, and artificial intelligence or machine learning. The full search strategy, including Boolean operators, platforms, and exact search dates, is provided in Supplementary Material S1. No language restrictions were applied. Preprints and abstract‐only conference reports were excluded because peer review and complete methodological reporting were considered necessary for reliable assessment of diagnostic performance, validation strategy, and risk of bias in AI‐based diagnostic studies. Reference lists of included studies were screened manually to identify any peer‐reviewed full‐text publications not captured by the primary database searches. Following expansion and rerunning of the primary search strategy, all included studies were identified through the four primary databases, and supplementary reference list screening did not identify any additional eligible studies.

### 2.4 Study Selection

**FIGURE 1:**
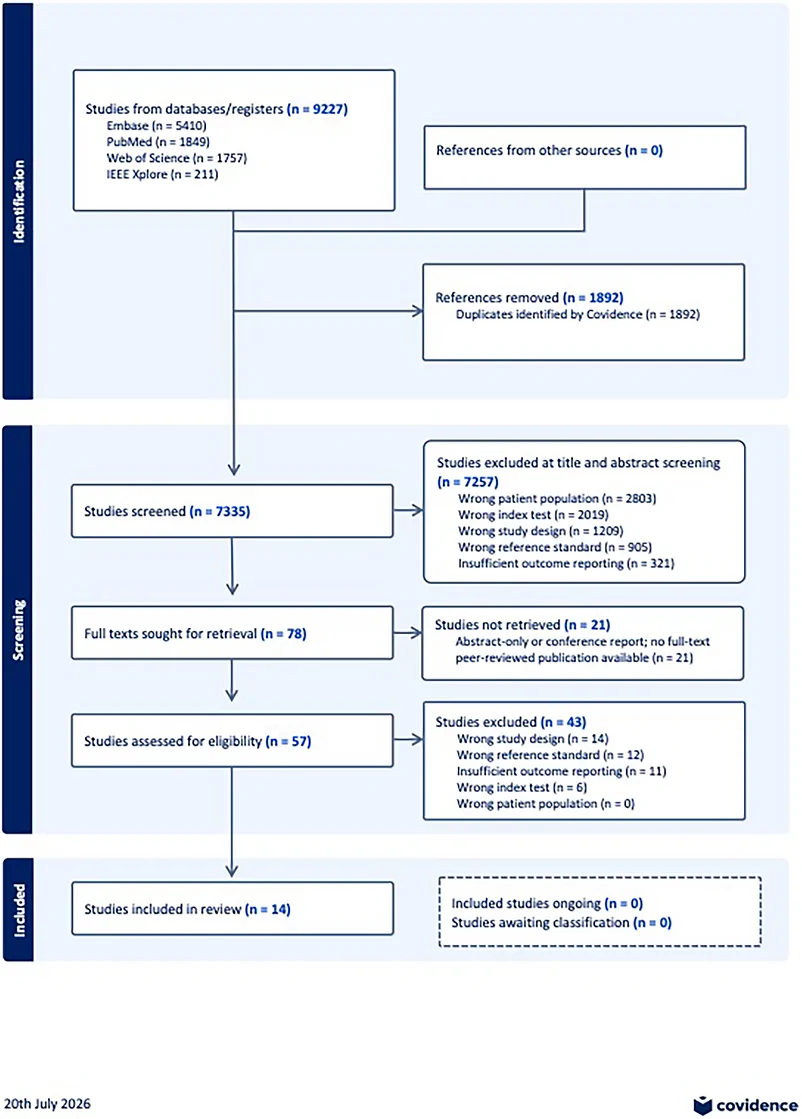
PRISMA diagram.

Two reviewers independently screened titles and abstracts for eligibility, followed by full‐text review of potentially relevant articles. Disagreements were resolved by consensus or, when required, adjudication by a third reviewer. Reasons for exclusion at the full‐text stage were recorded and are summarized in the PRISMA flow diagram (Figure 1).

### 2.5 Data Extraction

Data were extracted independently by two reviewers using a piloted standardized form, capturing study setting and population, echocardiographic inputs, reference standards, model characteristics and validation strategy, and diagnostic performance (AUC and other reported accuracy measures), including indeterminate and failure rates. Studies were required to report sufficient data to derive diagnostic performance estimates, including AUC or sufficient data to construct a 2×2 contingency table. Studies reporting only narrative or qualitative performance descriptions without extractable metrics were excluded. Disagreements were resolved by consensus/third reviewer, and data were managed in Covidence.

### 2.6 Risk of Bias and Applicability

Methodological quality and applicability were assessed independently by two reviewers using QUADAS‐3 [27]. Domain‐level risk‐of‐bias judgements were assigned as Low, High, or Unclear across four domains: patient selection, index test, reference standard, and flow and timing. Applicability concerns were assessed separately for patient selection, index test, and reference standard. Signaling questions and decision rules were adapted for AI diagnostic‐accuracy studies, with AI‐specific concerns including data leakage, label circularity, model transparency, and validation strategy incorporated into the relevant domain judgements rather than treated as a separate domain. Disagreements were resolved by consensus, with third‐reviewer adjudication where required. Full signaling questions, decision rules, and study‐level judgements are provided in Methods S3 and Table S4.

### 2.7 Synthesis Methods

Given anticipated heterogeneity in reference standards, target conditions, echocardiographic inputs, AI methodologies, and outcome reporting, we prespecified criteria for quantitative pooling and narrative synthesis.

#### 2.7.1 Invasive Reference Standard Studies

Random‐effects meta‐analysis of AUCs was planned only if at least three independent cohorts used harmonized invasive thresholds and reported sufficient uncertainty. These criteria were not met, as studies differed in invasive targets (PCWP, LVEDP, or relaxation indices), thresholds, populations, and AI index tests, with incomplete variance reporting. Results were therefore synthesized narratively, focusing on diagnostic discrimination and clinical context.

#### 2.7.2 Guideline‐Based LV Diastolic Dysfunction

Random‐effects pooling was planned when at least three studies assessed the same guideline‐based outcome and reported uncertainty estimates. This was not feasible due to heterogeneity in outcome definitions, performance metrics, confidence interval reporting, and handling of guideline‐indeterminate cases. Accordingly, results were summarized descriptively by clinical setting, model type, and reference definition.

#### 2.7.3 Parameter‐Level Replication

For replication of individual diastolic parameters (e.g., E/e′, e′ velocity, tricuspid regurgitation velocity, left atrial volume index), pooling was prespecified only when identical thresholds and uncertainty estimates were available across ≥3 cohorts. As thresholds and reporting were heterogeneous, parameter‐level results were summarized narratively and in tabular form.

#### 2.7.4 Summary Diagnostic Performance Metrics

As formal quantitative meta‐analysis was inappropriate, we performed a complementary descriptive summary of diagnostic discrimination across studies reporting AUCs. Studies were grouped into three synthesis strata: (1) invasive hemodynamic reference studies (PCWP or LVEDP as reference standard); (2) guideline‐label agreement studies (ASE/EACVI classification or expert consensus as reference); and (3) parameter‐level replication studies (discrimination for individual diastolic thresholds). These strata represent distinct analytical tasks rather than mutually exclusive publications. Stratum assignments were therefore made at the analytic‐arm level, allowing individual studies to contribute to more than one stratum where they reported multiple clinically distinct analyses. Within each stratum, AUCs are described with reference to validation approach (internal vs. external), cohort size, and population. Because the strata represent non‐equivalent clinical tasks—filling pressure prediction, label replication, and measurement automation respectively—no single summary statistic is reported across or within strata. Training‐only metrics, segmentation‐only outcomes, and studies reporting accuracy without ROC analysis were excluded from the AUC summary. Results are presented by synthesis stratum and described narratively.

### 2.8 Effect Measures and Data Handling

AUCs with 95% confidence intervals were extracted where reported. When studies included multiple cohorts (e.g., internal and external validation), these were treated as separate analytic arms. For the descriptive AUC summary, results are presented by synthesis stratum (invasive reference, guideline‐label agreement, parameter‐level replication) as specified in Section 2.7.4. Sensitivity and specificity were extracted at study‐defined operating points, and reported performance metrics were cross‐checked against available confusion matrices where possible. For each reported performance estimate, the denominator was defined as the sample on which that estimate was evaluated rather than the total study sample. Training and tuning samples were therefore excluded from validation denominators, while internal test, external test, and prospective evaluation cohorts were reported separately. For repeated cross‐validation designs, the aggregate out‐of‐fold evaluation sample or per‐iteration held‐out test sample was reported, as appropriate to the study design.

### 2.9 Indeterminate Classifications and Feasibility

We extracted rates of guideline‐based indeterminate classifications and the proportion of examinations successfully classified by AI systems. Feasibility metrics included view acquisition success, parameter measurement yield, and exclusions due to inadequate image quality. Where probabilistic grading, fallback models, or imputation strategies were applied, these were analyzed qualitatively.

### 2.10 Heterogeneity and Certainty of Evidence

Between‐study heterogeneity was assessed qualitatively by examining reference standards, thresholds, echocardiographic inputs, clinical settings, validation strategies, and risk‐of‐bias domains. Certainty of evidence was judged narratively, considering risk of bias, directness, consistency, precision, and extent of external validation, **without application of a formal grading scheme**. A quantitative summary of heterogeneity across included studies, including ML model type, number of echocardiographic input parameters, and analytic cohort sizes, is provided in Table S6.

## 3 Results

### 3.1 Study Selection and Characteristics

The database search identified 9227 records, of which 7335 remained after duplicate removal. Following title and abstract screening, 57 full‐text articles were assessed, and 14 studies met inclusion criteria (Figure 1) [13, 14, 15, 16, 17, 18, 19, 20, 21, 22, 23, 24, 25, 26].

Included studies were published between 2018 and 2026 and comprised echocardiographic examinations across Asia, Europe, and North America, spanning outpatient, inpatient, and emergency department/POCUS settings. Where public datasets (EchoNet‐Dynamic, CAMUS) were used, these contributed to technical components such as segmentation and view classification rather than direct clinical validation of diastolic dysfunction assessment. A cohort‐level breakdown specifying whether each reported sample represents a training, internal validation, external validation, or clinical test set is provided in Table S5. Sample sizes ranged from small proof‐of‐concept cohorts (44 patients [16]) to large multicohort validations (up to 31,241 examinations [13]). Study characteristics and index test details are summarized in Tables 1 and 2.

**TABLE 1:** Characteristics of included studies.

First author (year)	Country / setting	Population	Echo modality / views	Reference standard	Study design	Training / development set, *n*	Validation / test set, *n*	Primary outcomes studied
Cai (2024) [23]	China, hospital echo lab, external public datasets (US/FR)	Internal development: 862 + 239 echo studies; External: EchoNet‐Dynamic & CAMUS (public datasets)	Multi‐modality input: 2D echo videos + Doppler, six views auto‐selected for LVDF	ASE 2016 LV diastolic function algorithm, expert‐defined LVDF grades (decision‐tree/“hard” and probabilistic “soft” criteria)	Retrospective development across two internal cohorts	Internal DL development: 862 echocardiograms; ML correction model: 78 patients	Internal test: 239 echocardiograms**;** 212 evaluable for LVDF grading. External technical validation: Epiq‐7C 154; Doppler subset 74. EchoNet‐Dynamic 10,030 and CAMUS 500 used for LVEF/technical validation only	Develop a fully automated DL/ML workflow (MMnet) for guideline‐based LVDF grading; primary metrics: segmentation Dice, measurement MAE, and LVDF grading accuracy
Chen (2023) [22]	China, Multi‐center hospital echo labs	Development: 1304 (view‐classifier), 2238 (2D/Doppler segmentation), 2150 (single‐view classifiers). External test: 388 prospective studies	Multiview (auto view‐ID + 2D/Doppler segmentation), also single‐view (strain‐based / video‐based)	Two experts, guideline‐based LVDF detection and grading used as reference in 2150‐case training and 388‐case external test set	Retrospective development + prospective external testing	View classification: 1304 studies; 2D segmentation: 2238 studies; Doppler segmentation: 2198 images; single‐view LVDF models: 2150 studies	External prospective test: 388 studies	Build AI‐assisted system for LVDF detection and grading; primary outcomes, accuracy versus experts on external set
Gottlieb (2024) [18]	United States; single academic ED (POCUS)	209 enrolled (after exclusions) adult ED patients (≥45 y) at risk	POCUS PLAX, PSAX, A4C acquired; AI computed EF, E/A, septal & lateral e′	Expert consensus (two blinded physicians) for diastolic dysfunction (pre‐specified criteria: Diastolic dysfunction: E:A <0.8, or ≥2 of: septal e′ <7 cm/s, lateral e′ <10 cm/s, E:e′ >14**, or** LAVi >34 mL/m^2^ **.)**	Prospective observational diagnostic accuracy study	Not applicable — pre‐existing embedded vendor AI; no model training performed in study	Prospective clinical evaluation: 209 patients (220 enrolled; 11 excluded for inadequate images)	Determine diagnostic accuracy of AI for systolic & diastolic dysfunction versus expert reviewers
Gruca (2023) [16]	United States; single tertiary center; cath + TTE within 24 h	Total 294 (training 100; validation 194)	LA‐focused apical 4‐chamber; 2D speckle‐tracking LA strain; ML on full LAS time‐curve (LASi)	Invasive LVEDP ≥15 mmHg; compared head‐to‐head with peak LAS and 2016 ASE/EACVI algorithm	Retrospective development & hold‐out validation	100 patients	Internal hold‐out validation: 194 patients	Develop/validate LASi for detecting elevated LVEDP
Jiang (2022) [24]	Canada, academic echo lab	28,986 (train 23,188; internal validation 5798)	Structured echo parameters (2D + Doppler); tabular ML (no end‐to‐end video)	Non‐invasive ASE/EACVI 2016 diastolic grading (indeterminate excluded)	Retrospective development & internal validation	23,188 studies	Internal validation: 5798 studies	Create a continuous LV diastolic function score aligned to ASE/EACVI grades and identify thresholds for abnormal DF and elevated filling pressure (non‐invasive reference)
Karuzas (2025) [20]	Lithuania; single‐center outpatient TTE	302 adults with suspected LV diastolic dysfunction	2D TTE; fully automated measurements (Ligence Heart) including E, A, e′, E/A, LAVi, TR Vmax	Expert manual measurements with ASE/EACVI‐based LVDF grading (non‐invasive)	Prospective observational study	Not reported / not performed in study — pre‐existing commercial model	Prospective clinical evaluation: 302 patients	Assess feasibility and accuracy of automated LVDF assessment versus expert interpretation
Pandey (2021) [19]	Multicenter, USA‐led with international cohorts (US, Japan, Korea, Hungary)	Derivation *n* = 1242; external cohorts: hemodynamic *n* = 84, clinical outcomes n = 219, TOPCAT *n* = 518, pooled RELAX‐HF/NEAT‐HFpEF *n* = 346	Comprehensive TTE incl. 2D/Doppler and strain features used for model; routine echo parameters as inputs	Invasive LV filling pressure: PCWP on RHC or LV pre‐A pressure on LHC >15 mmHg for hemodynamic validation; guideline LVDD grading (ASE 2016) for comparison	Model development with external validation across hemodynamic, prognostic, and trial datasets	Derivation cohort: 1242 patients	External invasive hemodynamic validation: 84; external clinical‐outcome validation: 219; TOPCAT: 518; pooled RELAX‐HF/NEAT‐HFpEF: 346	Develop DeepNN to phenotype LVDD, detect elevated filling pressures, and stratify risk/outcomes beyond guideline grades
Park (2024) [15]	Republic of Korea; model developed on multi‐hospital AI‐Hub dataset	External evaluation *n* = 200: 167 adults with suspected LVDD (NYHA ≥II, LVEF ≥50%) plus 33 normal controls. key exclusions included AF, significant mitral disease, HCM, constriction, bundle branch block, pacing	Automated view selection and measurements from A4C and A2C B‐mode, PW MV inflow (E, A, DT), septal TDI (e′), CW TR Vmax; LA volume index and LA reservoir strain (LARS) derived from apical views	ASE/EACVI 2016 guideline‐directed LVDF detection and grading using AI‐derived versus manually derived measurements by an independent observer, clinician's prior report captured for comparison	Algorithm development with external diagnostic and prognostic validation	Multi‐hospital AI‐Hub development dataset: n not reported in main article	External diagnostic/prognostic evaluation: 200 examinations; separate external Mayo Doppler validation: 100 examinations	Feasibility/accuracy of automated LVDF pipeline (view ID, parameter measurement), agreement with manual guideline grading, discrimination for abnormal parameters, and prognostic value of AI‐derived LVDF grade and LARS for clinical outcomes
Qu (2023) [14]	China; single‐center hospital dataset	Diastolic dataset 286 post‐CABG patients (77 with diastolic dysfunction), separate segmentation dataset 91 patients (8816 A4C images)	Single‐view apical 4‐chamber 2D TTE, LV/LA U‐Net segmentation → morphological 1‐D curves (area/length/width) → FFANet classifier	Clinician‐assigned diastolic dysfunction versus normal, non‐invasive labels, guideline basis not specified, no catheter standard	Method development, retrospective, internal split (8:2 for classification; 9:1 for segmentation)	Diastolic classifier: ≈229 patients (80% of *n* = 286); segmentation model: ≈82 patients (90% of *n* = 91)	Internal diastolic test/validation: ≈57 patients (20% of *n* = 286); segmentation validation: ≈9 patients (10% of *n* = 91)	Propose and test FFANet to classify diastolic function using A4C morphology alone; primary outcome = diagnostic performance (accuracy/AUC)
Salem Omar (2018) [17]	USA, academic center	174 total (train 130; test 44 with invasive hemodynamics)	2D speckle‐tracking echocardiography (LV/LA deformation features from apical views)	Non‐invasive label: elevated LVFP by E/e′ ≥13, invasive subset validation: PCWP ≥18 mmHg (RHC)	Retrospective development letter	130 patients	Held‐out test/invasive hemodynamic cohort: 44 patients	Develop ML model to detect elevated LV filling pressure from 2D STE
Xu (2024) [21]	USA, single center, TTE within 24 h of elective cath	460 consecutive adults (2008–2010)	Routine TTE parameters (2D/Doppler) as tabular features: E, A, e′, E/e′, LA volume, TR, MV DT, and so forth plus clinical and NT‐proBNP (no raw images)	Invasive LVEDP >20 mmHg (LHC) for primary task, invasive tau >45 ms for secondary task	Retrospective ML study, 20‐iteration train/validate/test workflow with random 9:1 train–test and 9:1 train–val splits	Per iteration: ≈373 training + ≈41 tuning/validation patients	Per iteration: ≈46 internal test patients; random splitting repeated 20 times	Develop ML models to predict elevated LVEDP and tau from non‐invasive echo + clinical/biomarker data
Tromp (2022) [13]	Multicenter Asia development and internal test External validation in Canada Taiwan USA	Train 1145 echocardiograms from 1076 patients Internal test 406 echocardiograms External Canada 1029 Taiwan 31,241 USA EchoNet 10,030 Prospective variability 142 echocardiograms	End to end workflow on 2D TTE videos and Doppler modalities including A4C A2C PLAX pulsed and continuous wave Doppler and tissue Doppler imaging with automated LVEF E e′ e′ lateral and E/e′	Expert manual sonographer measurements used as ground truth across cohorts. Diagnostic thresholds used for reporting included LVEF less than 40 percent E/e′ at least 13 and e′ lateral less than 10 cm/s. No invasive hemodynamics	Algorithm development with internal test set and multicohort external validation plus independent prospective reproducibility study	1145 echocardiograms	Internal test: 406; external Canada: 1029; external Taiwan: 31,241; prospective evaluation: 142. EchoNet‐Dynamic: 10,030, used for LVEF validation only	Develop and validate a fully automated deep learning workflow for view classification segmentation and measurement of systolic and diastolic function versus human experts
Khan (2026) [25]	Multinational, multicenter; 6 tertiary centers in Norway, USA, South Korea, Spain, and Japan	250 patients undergoing echocardiography and invasive catheterization; 108 prospectively and 142 retrospectively enrolled	Comprehensive TTE; 64 candidate clinical, biomarker, and echocardiographic variables, reduced to 47 candidate predictors	Invasive LVFP: PAWP or LV pre‐A pressure >15 mmHg, or LVEDP >16 mmHg	Multicenter cohort; nested cross‐validation	Approx. 200 patients per outer training fold; inner 4‐fold CV for feature selection/hyperparameter tuning	Approx. 50 per held‐out outer test fold; *n* = 250 aggregate out‐of‐fold evaluation	Classification of normal versus elevated LVFP; comparison with 2016 ASE/EACVI and 2022 EACVI algorithms; feature importance
Nakamura (2026) [26]	Japan, multicenter; 3 hospitals	956 patients undergoing TTE and RHC within 1 week	Comprehensive routine TTE measurements	Invasive RHC: PAWP ≥18 mmHg	Retrospective multicenter diagnostic accuracy study	Development cohort from 2 hospitals, *n* = 621; 10‐fold CV/Bayesian hyperparameter tuning	Independent third‐hospital external test cohort, *n* = 335	Explainable XGBoost prediction of elevated LVFP; comparison with guideline algorithm; SHAP interpretation

Abbreviations: 2D, two‐dimensional; A2C, apical two‐chamber view; A4C, apical four‐chamber view; AI, artificial intelligence; ASE, American society of echocardiography; AUC, area under the receiver operating characteristic curve; CABG, coronary artery bypass grafting; CAMUS, cardiac acquisitions for multi‐structure ultrasound segmentation dataset; cath, cardiac catheterization; CV, cross‐validation; CW, continuous‐wave Doppler; DeepNN, deep neural network; DL, deep learning; E and A, mitral inflow early‐ and late‐diastolic velocities; E/A, ratio of E to A; E/e′, ratio of E to e′; e′, mitral annular early diastolic tissue velocity; EACVI, European association of cardiovascular imaging; EchoNet‐Dynamic, public echocardiography video dataset; ED, emergency department; EF, left ventricular ejection fraction; HF, heart failure; LA, left atrium; LAESV, left atrial end‐systolic volume; LARS, left atrial reservoir strain; LAS, left atrial strain; LASi, left atrial strain index; LAVi, left atrial volume index; LHC, left‐heart catheterization; LV, left ventricle; LVDD, left ventricular diastolic dysfunction; LVDF, left ventricular diastolic function; LVEDP, left ventricular end‐diastolic pressure; LVFP, left ventricular filling pressure; MAE, mean absolute error; MMnet, hybrid multi‐task model name; MV DT, mitral inflow deceleration time; NEAT‐HFpEF, nitrate's effect on activity tolerance in HFpEF trial; NT‐proBNP, N‐terminal pro–B‐type natriuretic peptide; PCWP, pulmonary capillary wedge pressure; PLAX, parasternal long‐axis view; POCUS, point‐of‐care ultrasound; PSAX, parasternal short‐axis view; PW, pulsed‐wave Doppler; RELAX‐HF, trial of phosphodiesterase‐5 inhibition in HFpEF; RHC, right‐heart catheterization; STE, speckle‐tracking echocardiography; TDI, tissue Doppler imaging; TOPCAT, aldosterone antagonist therapy for adults with HFpEF trial; TR, tricuspid regurgitation; TR Vmax, peak tricuspid regurgitation velocity; TTE, transthoracic echocardiography.

**TABLE 2:** AI/ML model details.

First author (year)	Index test / model	Preprocessing	Model and training (architecture, split/CV)	Label source (ASE 2016 vs. invasive; adjudication)	Thresholding / operating point selection
Cai (2024) [23]	MMnet multi‐task DL + RF; view classification, LV/LA segmentation, keypoints, Doppler peaks (E, A, e′, TRmax)	Auto view selection, chamber segmentation, ED/ES detection, Simpson volumes, Doppler peak extraction; 256×256 inputs	ResNet50_tiny multi‐head; 10‐fold CV; Adam (1e−4), batch 8; inference 2–3 s	ASE/EACVI 2016 expert labels; no invasive	Fixed ASE cutoffs + ROC; hard grades and probabilistic mapping
Chen (2023) [22]	AI‐assisted LVDF workflow: multiview rule‐based grading + single‐view SVM (strain) and S3D‐G CNN	View classifier (Xception), U‐Net chamber/Doppler segmentation; automated Doppler, volumes, strain	4:1 split; Adam ∼150 epochs; external prospective test (*n* = 388)	Two experts (ASE 2016); adjudicated; no invasive	Rule‐based multiview; ROC‐derived cutoffs for strain SVM
Gottlieb (2024) [18]	Vendor AI (Mindray TE X): Auto‐EF, Auto‐DFR	Standard POCUS views; AI auto‐measurement (no TR)	Proprietary embedded model; prospective testing only	Two expert reviewers; consensus; no invasive	ASE‐based rule definition of dysfunction
Gruca (2023) [16]	LAS index (PLS regression on LA strain curve)	A4C LA strain; time‐normalized (25 points); smoothing; quality exclusion	PLS (*n* = 100 train), validated *n* = 194; 5‐fold CV + 1000× resampling	Invasive LVEDP ≥15 mmHg	ROC‐selected cut‐point; optional indeterminate zone
Jiang (2022) [24]	Tabular ML (SVM/DT/XGB/DNN); R‐DNN continuous score	Retrospective echo variables; software reclassified ASE 2016; indeterminate excluded	80/20 split; grid search; transfer learning for R‐DNN	Guideline‐based labels (non‐invasive)	ROC cutoffs (Youden); score >0.91 normal, >0.65 elevated filling pressure
Karuzas (2025) [20]	Fully automated CNN (“Ligence Heart”)	Auto view, cycle, frame, Doppler extraction; prospective single‐center	Commercial model; no disclosed architecture; prospective test	Single expert (ASE 2016); no invasive	Fixed ASE cutoffs; grade‐level precision/recall
Pandey (2021) [19]	DeepNN on tabular features after unsupervised TDA phenogrouping	Echo variables only; similarity network; no image input	AutoML; derivation *n* = 1242; external invasive/outcomes validation	TDA‐derived high/low risk groups; invasive only for validation	Default model boundary; AUC reported
Park (2024) [15]	Fully automated pipeline incl. LA strain	Auto view selection; chamber/Doppler segmentation; LARS tracking	Multi‐center dev (∼30k exams); prospective ext test *n* = 200	ASE 2016 grading; manual comparator; no invasive	Guideline cutoffs; standard ASE grading logic
Qu (2023) [14]	FFANet (1D ResNet‐18 with frequency attention)	A4C cine; LV/LA segmentation; area curves → 6×224 signals	8:2 split; Adam 0.001; no CV	Clinician label (DD vs. normal); non‐invasive	Sigmoid 0.5 threshold
Salem Omar (2018) [17]	Ensemble (RF + ANN + SVM; majority vote) on strain features	Speckle‐tracking (14 features); feature selection	10‐fold CV; train 130/test 44	Non‐invasive E/e′ ≥13; invasive PCWP ≥18 mmHg validation	Majority vote (≥2/3 models)
Xu (2024) [21]	Tabular ML (LR best) for LVEDP and tau	Collinearity filtering; imputation; scaling	Repeated 9:1 splits ×20; grid search	Invasive LVEDP >20 mmHg; tau >45 ms	Physiologic cutoffs; AUROC reported
Tromp (2022) [13]	Fully automated DL workflow (view, segmentation, Doppler CNNs)	DICOM sector cropping; ECG phase detection; quality gating	Trained on 55,487 images from 1145 echocardiograms (ATTRaCT); multi‐national external validation	Expert manual measurements; no invasive	Predefined clinical cutpoints; no tuned threshold
Khan (2026) [25]	Eight supervised ML classifiers: XGBoost, support vector classifier, linear SVC, gradient boosting, logistic regression, k‐nearest neighbors, random forest, and decision tree	64 initial clinical, biomarker and echocardiographic variables; prespecified filtering reduced these to 47 candidate features;	Nested 5‐fold CV: outer held‐out test folds; inner 4‐fold CV for recursive feature selection; grid search for hyperparameter optimization	Invasive elevated LVFP: PAWP/pre‐A >15 mmHg or LVEDP >16 mmHg	Binary classification of normal versus elevated LVFP; all ML models produced determinate classifications
Nakamura (2026) [26]	Two XGBoost models with SHAP explainability	Model 1: E/A, e′, E/e′, LAVI, TRV. Model 2: Model 1 variables plus maximum IVC diameter, LV end‐diastolic dimension, LA dimension, and LVEF selected using SHAP importance	*n* = 621 development cohort; 10‐fold CV with Bayesian hyperparameter optimization; final models tested unchanged in independent *n* = 335 external cohort	PAWP ≥18 mmHg by RHC	Binary probability and classification of elevated LVFP; SHAP used for global and patient‐level interpretation

Abbreviations: AI, artificial intelligence, A4C, apical four‐chamber view; ANN, artificial neural network; ASE, American society of echocardiography; AUROC, area under the receiver operating characteristic curve; AutoML, automated machine learning; CNN, convolutional neural network; CV, cross‐validation; DD, diastolic dysfunction; DICOM, Digital Imaging and Communications in Medicine; DL, deep learning; DNN, dense neural network; DT, decision tree; E, early diastolic transmitral inflow velocity; E/e′, ratio of mitral inflow E to tissue Doppler e′; e′, early diastolic mitral annular velocity; EACVI, European association of cardiovascular imaging; ECG, electrocardiogram; ED, end‐diastole; ES, end‐systole; LA, left atrium; LARS, left atrial reservoir strain; LAS, left atrial strain; LVDF, left ventricular diastolic function; LVEDP, left ventricular end‐diastolic pressure; LVEF, left ventricular ejection fraction; LVFP, left ventricular filling pressure; ML, machine learning; PCWP, pulmonary capillary wedge pressure; PLS, partial least squares regression; POCUS, point‐of‐care ultrasound; R‐DNN, regression dense neural network; RF, random forest; ROC, receiver operating characteristic; S3D‐G, separable three‐dimensional convolutional neural network; STE, speckle‐tracking echocardiography; SVM, support vector machine; TDA, topological data analysis; TRmax, peak tricuspid regurgitation velocity; U‐Net, encoder‐decoder segmentation network; XGB, XGBoost (gradient‐boosted decision trees).

Across studies, echocardiographic inputs varied from raw multiview 2D cine plus Doppler (Cai et al. [23]; Chen et al. [22]; Tromp et al. [13]; Park et al. [15]), to commercial automated measurement software tested prospectively (Karužas et al. [20]), to tabular machine learning using routine report variables with or without biomarkers (Jiang et al. [24]; Xu et al. [21]; Pandey et al. [19]), to single‐view A4C morphology (Qu et al. [14]), and speckle‐tracking deformation features (Salem Omar et al. [17]; Gruca et al. [16]).

### 3.2 Reference Standards and Clinical Contexts

Included studies clustered naturally by reference standard and clinical intent. Five studies primarily evaluated AI against invasive hemodynamic reference standards, while Salem Omar et al. [17] additionally reported a small exploratory invasive substudy of a model trained using a non‐invasive surrogate label [16, 19, 21, 25, 26]. Across these studies, invasive reference standards and target definitions varied considerably, including PCWP or LV pre‐atrial pressure >15 mmHg, LVEDP ≥15 mmHg, LVEDP >16 mmHg, LVEDP >20 mmHg, tau >45 ms, and PCWP ≥18 mmHg.

Beyond the five primary invasive‐reference studies, eight studies contributed to the non‐invasive reference stratum, including guideline‐based or expert‐defined LVDF classification and one surrogate‐label analysis [13, 14, 15, 18, 20, 22, 23, 24]. Three studies contributed to the parameter‐level replication stratum by evaluating automated measurement of individual diastolic thresholds against expert manual measurements [13, 15, 23]. Because strata were assigned at the analytic‐arm level, Cai et al. [23] and Park et al. [15] contributed to both guideline‐grade agreement and parameter‐level replication, while Salem Omar et al. [17] was classified as a surrogate‐label study with its exploratory invasive substudy reported descriptively. These assignments are detailed in Table 1 and S8.

### 3.3 AI Approaches Evaluated

AI methodologies varied substantially across studies, reflecting differences in clinical intent, echocardiographic inputs, and validation strategy. Fully automated multiview deep learning systems performed view selection, chamber segmentation, Doppler analysis, and diastolic grading in an end‐to‐end manner (Cai et al. [23]; Chen et al. [22]; Tromp et al. [13]; Park et al. [15]). Hybrid approaches combined deep learning–based measurement extraction with rule‐based or tabular machine‐learning classifiers trained on structured echocardiographic variables (Jiang et al. [24]; Xu et al. [21]; Pandey et al. [19]). Additional tabular machine‐learning approaches used structured clinical, biomarker, and echocardiographic variables to predict invasively defined elevated filling pressures (Khan et al. [25]; Nakamura et al. [26]). Other strategies included commercial automated measurement software (Karužas et al. [20]), device‐embedded POCUS AI (Gottlieb et al. [18]), and single‐view morphology‐only classification (Qu et al. [14]). This methodological heterogeneity—spanning four end‐to‐end deep learning pipelines, six tabular ML or ensemble approaches, two hybrid systems, and two commercial or embedded platforms—informed the synthesis strategy and precluded a single pooled estimate of diagnostic performance (Table S6).

### 3.4 Diagnostic Performance Versus Invasive Hemodynamic Reference

Five studies primarily compared AI‐derived echocardiographic assessments with invasive hemodynamic measurements (Pandey et al. [19]; Gruca et al. [16]; Xu et al. [21]; Khan et al. [25]; Nakamura et al. [26]), together with one small exploratory invasive substudy from a model trained on non‐invasive surrogate labels (Salem Omar et al. [17]). Quantitative meta‐analysis was not performed because of incompatible reference standards, thresholds, and incomplete reporting of variance estimates (Table 3).

**TABLE 3:** Model performance and outcomes.

First author (year)	Performance metric type	Validation / test denominator	AUC (95% CI)	Sensitivity (95% CI) Specificity (95% CI)	TP / FP / FN / TN	Indeterminate / failure rate (%)	Comparator and key result
**Stratum 1: Invasive hemodynamic reference studies**							
Pandey (2021) [19]—invasive hemodynamic validation	AUC (invasive reference)	External invasive hemodynamic cohort, *n* = 84	DeepNN 0.883 (CI NR); ASE/EACVI 0.676 (CI NR) in overall cohort	NR	NR	ASE/EACVI indeterminate 18%–43% across validation settings; DeepNN 0%	DeepNN superior to ASE/EACVI grading for detection of invasively elevated filling pressure in the full cohort
Pandey (2021) [19]—clinical outcome validation	Prognostic discrimination / external clinical validation	External prospective outcome cohort, *n* = 219	NR	NR	NR	DeepNN 0%	High‐risk phenogroup associated with greater risk of death and/or HF hospitalization; secondary clinical validation rather than diagnostic validation
Pandey (2021) [19]—trial‐cohort outcome validation	Prognostic / clinical phenotype validation	TOPCAT *n* = 518; pooled RELAX‐HF/NEAT‐HFpEF *n* = 346	NR	NR	NR	DeepNN 0%	High‐risk phenotype associated with adverse outcomes, greater biomarker burden and impaired exercise performance; in TOPCAT, spironolactone associated with lower risk in the high‐risk phenogroup
Xu (2024) [21]	AUC (invasive reference)	Internal test ≈46 patients per iteration; repeated over 20 random splits	LVEDP >20 mmHg: LR 0.761 (0.725–0.796); tau >45 ms: GB 0.832 (0.700–0.964)	NR	NR	NR	Internal algorithm comparison only; LR best for LVEDP and GB best for tau; no external validation
Gruca (2023) [16]	Accuracy; sensitivity/specificity (invasive reference)	Internal hold‐out validation, *n* = 194	NR	0.80 / 0.77 (CI NR)	93 / 18 / 23 / 60	ASE/EACVI indeterminate 37/194 (19.1%). LASi has no indeterminate result by default; with a predefined near‐zero LASi band sized to 37 cases, accuracy improved to 0.87	LASi accuracy 0.87 with indeterminate band; outperformed peak LA strain and was comparable to ASE/EACVI grading
Salem Omar (2018) [17]—surrogate‐label model evaluation	AUC (non‐invasive surrogate label)	Main model‐development cohort: training *n* = 130; held‐out test *n* = 44	10‐fold CV: train 0.847, test 0.868 with 14 features; train 0.853, test 0.881 with 11‐feature subset (all CI NR)	NR	NR	NR	Ensemble STE+ML predicted E/e′ ≥13; these AUCs reflect surrogate‐label performance and not invasive hemodynamic validation
Salem Omar (2018) [17]—invasive substudy	Sensitivity (invasive reference)	Invasive test subset, *n* = 44	NR	Sensitivity 80% (CI NR) for PCWP ≥18 mmHg; specificity NR	NR	NR	Exploratory invasive comparison; no invasive‐reference AUC reported
Khan (2026) [25]	AUC (invasive reference)	*n* = 250 aggregate out‐of‐fold predictions from 5 held‐out test folds	0.87 (CI NR)	Sensitivity 75%; specificity 92%	NR	0%	2016 ASE/EACVI: AUC 0.80, accuracy 81%, 13% unclassified; 2022 EACVI: AUC 0.76, accuracy 78%, 3% unclassified. Across all 8 ML models, accuracy 82–86% and AUC 0.80–0.89
Nakamura (2026) [26]—Model 1	AUC (invasive reference)	*n* = 335 independent external test	0.81 (0.75–0.87)	Sensitivity 82%; specificity 71%	NR	0%	Outperformed the guideline algorithm; in the guideline‐classifiable subgroup, AUROC 0.82 (0.73–0.92) versus 0.72 (0.60–0.83), *p* = 0.020
Nakamura (2026) [26]—Model 2	AUC (invasive reference)	*n* = 335 independent external test	0.84 (0.79–0.89)	Sensitivity 77%; specificity 79%	NR	0%	Best overall model; in the guideline‐classifiable subgroup, AUROC 0.83 (0.75–0.91) versus guideline algorithm 0.72, *p* = 0.016; AUROC 0.84 (0.78–0.91) in guideline‐indeterminate cases
**Stratum 2: Guideline‐label / expert agreement studies**							
Jiang (2022) [24]	AUC (guideline‐label agreement)	Internal validation, *n* = 5798	Abnormal LVDF 0.99 (CI NR); elevated filling pressure 0.99 (CI NR)	NR	NR	Continuous score classified 68.7% of guideline‐indeterminate cases as abnormal	98% accuracy versus ASE/EACVI grades; score correlated with grade severity; no external validation. Training *n* = 23,188
Qu (2023) [14]	AUC (expert‐label agreement)	Internal test/validation, ≈57 patients	0.922 (CI NR)	NR	NR	NR	FFANet outperformed comparator architectures; denominator inferred from reported 8:2 split of total *n* = 286; no external validation
Gottlieb (2024) [18]	Sensitivity/specificity (expert consensus)	Prospective clinical evaluation, *n* = 209	NR	91.9% (85.6–96.0) / 94.2% (87.0–98.1)	NR	11/220 enrolled (5.0%) excluded for inadequate image quality	LR+ 15.8; LR− 0.09; prospective POCUS evaluation
Chen (2023) [22]—single‐view strain/SVM internal validation	AUC (guideline‐label agreement)	Internal validation set; exact n not separately reported	Multiview accuracy 0.90 (CI NR) detection, 0.92 (CI NR) grading. Single‐view SVM AUC ∼0.94–0.96 (CI NR); 3D‐CNN AUC 0.81–0.89 (CI NR)	NR	NR	NR	ROC analysis of strain‐derived metrics and SVM combinations from a single A4C view; internal validation only and separate from the external prospective evaluation
Chen (2023) [22]—external prospective clinical evaluation	Accuracy (expert guideline‐label agreement)	External prospective test, *n* = 388	NR	NR	NR	NR	Multiview accuracy 0.90 for LVDD detection and 0.92 for grading; external comparison against two expert reviewers
Karužas (2025) [20]	Agreement / classification performance	Prospective clinical evaluation, *n* = 302	NR	Grade‐level recall: Grade 0 56.8%, Grade 1 77.4%, Grade 2 40.7%, Grade 3 42.9% (CI NR);	Grade 0 92.1%, Grade 3 99.7% (CI NR)	NR	Strong parameter agreement, including E r = 0.93 and LAVi r = 0.92; best classification at normal and severe grades, with lower recall for intermediate grades
**Stratum 3: Parameter‐level replication studies**							
Tromp (2022) [13]	AUC (parameter‐level replication)	Internal test *n* = 406; Canada *n* = 1029; Taiwan *n* = 31,241	E/e′ ≥13: Asia 0.96 (0.92–0.99), Canada 0.91 (0.88–0.94), Taiwan 0.91 (0.89–0.93); e′ lateral <10 cm/s: Asia 0.95 (0.88–0.99), Canada 0.88 (0.84–0.92), Taiwan 0.94 (0.93–0.95)	NR	NR	Confidence‐based quality gating used	Strong multicohort generalization for E/e′ and e′ threshold replication; lower measurement variability than expert sonographers
Park (2024) [15]	AUC (parameter‐level replication)	External clinical evaluation, *n* = 200	Septal e′ <7: 0.961; E/e′ >15: 0.966; TR Vmax >2.8: 0.967; LAVi >34: 0.977 (CIs NR)	NR	NR	NR	94% concordance with manual guideline grading; separate Mayo Doppler measurement validation *n* = 100
Cai (2024) [23]—parameter‐threshold replication	AUC (parameter‐level replication)	Internal test cohort, *n* = 239 overall; available‐case denominators varied by parameter	E/e′ >14: 0.992 (0.9774–0.9973); septal e′ <7: 0.990 (0.9713–0.9978); lateral e′ <10: 0.995 (0.9684–0.9978); TRmax >280: 0.977 (0.9559–0.9907)	NR	NR	Parameter‐specific missingness; available cases varied	High discrimination for reproducing manually measured guideline parameter thresholds
Cai (2024) [23]—final LVDF grading	Accuracy (guideline‐based grading)	LVDF grading cohort, *n* = 212	NR	NR	NR	NR	Hard‐rule grading accuracy 0.88; probabilistic soft grading accuracy up to 0.98

Abbreviations: ASE, American society of echocardiography; AUC, area under the receiver operating characteristic curve; AUROC, area under the receiver operating characteristic curve; CI, confidence interval; CNN, convolutional neural network; CV, cross‐validation; DeepNN, deep neural network; DF, diastolic function; E, early diastolic transmitral inflow velocity; E/e′, ratio of mitral inflow E to tissue Doppler e′ velocity; e′, early diastolic mitral annular velocity; EACVI, European Association of Cardiovascular Imaging; FN, false negatives; FP, false positives; GB, gradient boosting; HF, heart failure; HR, hazard ratio; LASi, left atrial strain index; LAVi, left atrial volume index; LR, logistic regression; LR−, negative likelihood ratio; LR+, positive likelihood ratio; LVDF, left ventricular diastolic function; LVEDP, left ventricular end‐diastolic pressure; LVEF, left ventricular ejection fraction; ML, machine learning; NR, not reported; PCWP, pulmonary capillary wedge pressure; STE, speckle‐tracking echocardiography; TN, true negatives; TP, true positives; TR, tricuspid regurgitation; TRmax, peak tricuspid regurgitation velocity.

Across these studies, AI models integrating multiple echocardiographic and clinical features demonstrated moderate‐to‐high discrimination for elevated filling pressures or abnormal relaxation. Performance gains relative to guideline‐based grading were most evident in cohorts containing guideline‐indeterminate cases, where AI systems generated determinate outputs and showed graded associations with invasively measured hemodynamics. Among invasive‐reference studies reporting ROC‐based performance, the primary analyses contributing to the synthesis yielded AUROCs ranging from 0.761 to 0.883. Secondary subgroup analyses and alternative models are presented descriptively but were not used to derive the reported range. These studies differed substantially in invasive targets, thresholds, populations, and validation approaches, and no summary statistic is reported across them.

Pandey et al. [19] evaluated a deep neural network trained on nine guideline‐derived echocardiographic variables in 84 patients undergoing catheterization, defining elevated filling pressure as PCWP or LV pre‐atrial contraction pressure >15 mmHg. In the full cohort, including guideline‐indeterminate cases, DeepNN showed higher discrimination than ASE/EACVI 2016 grading (AUROC 0.88 vs 0.67; *p* = 0.01), while performance was similar among guideline‐classifiable cases only (AUROC 0.89 vs. 0.83; *p* = 0.32), indicating that the benefit was driven largely by resolution of indeterminate cases rather than superior performance in straightforward cases. This within‐study subgroup difference is clinically important, it implies that AI adds diagnostic value predominantly in the patients guideline grading cannot classify, rather than replacing guideline assessment in straightforward cases. Predicted probabilities correlated with invasive filling pressures across the broader validation cohorts (*r* = 0.62, *p* < 0.001), with clear phenogroup separation (*p* = 0.004).

Gruca et al. [16] developed a machine‐learning–derived left atrial strain index (LASi) incorporating the full strain time curve and validated it in 194 patients with invasive LVEDP ≥15 mmHg. LASi identified elevated LVEDP with 79% accuracy (80% sensitivity, 77% specificity), outperforming peak strain and ASE/EACVI grading, which was indeterminate in 19.1% of cases. Applying a predefined indeterminate zone to the LASi score—sized to match the 19.1% guideline‐indeterminate rate—increased overall accuracy to 87%, demonstrating that within‐study performance varied meaningfully depending on whether a binary or three‐zone classification strategy was applied.

Xu et al. [21] evaluated tabular machine‐learning models integrating clinical variables, echocardiographic parameters, and NT‐proBNP in 460 patients undergoing elective catheterization. Performance varied substantially by target within the same study: logistic regression achieved the highest discrimination for elevated LVEDP >20 mmHg (AUROC 0.761), while gradient boosting performed best for impaired relaxation defined by tau >45 ms (AUROC 0.832). This within‐study divergence ‐ 0.761 versus 0.832 for two invasive targets in the same cohort—reflects the different hemodynamic information content of LVEDP elevation versus impaired relaxation. Feature importance analyses highlighted complementary contributions from hemodynamic surrogates, atrial size, Doppler timing indices, and NT‐proBNP.

Khan et al. [25] evaluated eight supervised machine‐learning classifiers in 250 patients from six tertiary centers across five countries, using invasively defined elevated LV filling pressure as the reference standard. Nested five‐fold cross‐validation separated model optimization from held‐out testing. Across models, accuracy ranged from 82% to 86% and AUROC from 0.80 to 0.89; logistic regression achieved an AUROC of 0.87, with 75% sensitivity, 92% specificity, and 86% accuracy. By comparison, the 2016 ASE/EACVI and 2022 EACVI algorithms achieved AUROCs of 0.80 and 0.76 and left 13% and 3% of patients unclassified, respectively, whereas all machine‐learning models generated determinate classifications. Feature importance highlighted complementary contributions from atrial strain, NT‐proBNP, and conventional Doppler parameters.

Nakamura et al. [26] developed two explainable XGBoost models in 621 patients and tested them unchanged in an independent external cohort of 335 patients, using PAWP ≥18 mmHg as the reference standard. Model 1 achieved an AUROC of 0.81 (95% CI 0.75–0.87), while the expanded SHAP‐informed Model 2 achieved 0.84 (95% CI 0.79–0.89), with 77% sensitivity, 79% specificity, and 79% accuracy. Both models outperformed the guideline algorithm among classifiable patients, while Model 2 retained an AUROC of 0.84 in guideline‐indeterminate cases. Both generated determinate classifications for all patients, demonstrating that performance and resolution of indeterminate cases were maintained in an independent external cohort.

Salem Omar et al. [17] developed an ensemble machine‐learning model using speckle‐tracking–derived deformation features to classify elevated filling pressure based on the non‐invasive surrogate E/e′ ≥13. Cross‐validated performance was high (AUC 0.85–0.88), with the 11‐feature subset performing comparably to the full 14‐feature model (AUC 0.881 vs. 0.868 on test). In a small exploratory invasive substudy (*n* = 44), the model identified 80% of patients with PCWP ≥18 mmHg. However, no AUC was reported for the invasive substudy, precluding direct comparison with the cross‐validated surrogate‐label performance.

### 3.5 Diagnostic Performance Versus Guideline‐Based Diastolic Function

Eight studies evaluated AI systems for either automated guideline‐based diastolic function classification or replication of expert echocardiographic measurements associated with diastolic assessment, predominantly using ASE or ASE/EACVI 2016 criteria as the reference standard, across echocardiography laboratory and emergency department settings (Table 3) (Cai et al. [23]; Chen et al. [22]; Tromp et al. [13]; Park et al. [15]; Jiang et al. [24]; Karužas et al. [20]; Gottlieb et al. [18]; Qu et al. [14]).

The most robust evidence came from large, multiview, fully automated systems with external or multicohort validation. Tromp et al. validated an end‐to‐end deep learning workflow for automated extraction and interpretation of systolic and diastolic echocardiographic parameters using expert manual measurements as the reference standard across cohorts from Asia (*n* = 406), Canada (*n* = 1,029), and Taiwan (*n* = 31,241), demonstrating consistently high discrimination for key diastolic indices, including AUCs of 0.96, 0.91, and 0.91 for E/e′ ≥13 across cohorts, with similarly strong performance for lateral e′ <10 cm/s [13]. In a prospective reproducibility substudy (*n* = 142), automated measurements showed lower variance than repeated expert sonographer measurements.

Cai et al. [23] evaluated a fully automated hybrid deep learning and machine‐learning pipeline (MMnet) performing multiview classification, chamber segmentation, Doppler extraction, and guideline‐based grading. In internal clinical testing, grading accuracy was 0.88 using strict ASE/EACVI thresholds and increased to 0.98 with probabilistic grading. The reported diastolic‐performance estimates were derived from internal testing only. External public datasets were used for technical validation of segmentation and LVEF components, and were not considered external clinical validation of diastolic targets. Chen et al. [22] reported similar performance in an external prospective cohort of 388 studies, with accuracies of 0.90 for detection and 0.92 for grading severity, and demonstrated preserved performance using single‐view fallback models when datasets were incomplete.

Park et al. [15] validated a multicenter automated framework in 200 studies, achieving 99.1% accuracy for view identification and 94.0% concordance with manual guideline grades, with strong agreement for individual diastolic parameters (Pearson r 0.90–0.96).

Evidence from tabular, commercial, single‐view, and point‐of‐care approaches was more heterogeneous. Jiang et al. [24] reported excellent internal discrimination (AUC 0.99) in an internal validation cohort of 5798 studies, following model development in 23,188 studies, although exclusion of indeterminate cases and lack of external validation limit generalizability. Karužas et al. [20] prospectively evaluated a commercial automated echocardiography platform in 302 adults, demonstrating strong correlations with expert measurements for core diastolic parameters, but reduced recall for intermediate grades. Qu et al. [14] demonstrated feasibility of single‐view apical four‐chamber morphology‐based classification in a post–coronary artery bypass graft cohort of 286 patients, using an 80:20 development/test split, with performance evaluated in an internal test set of approximately 57 patients; no external validation was performed.

In the emergency department, Gottlieb et al. [18] evaluated a device‐embedded point‐of‐care ultrasound AI system in 220 adults, achieving sensitivity of 91.9% and specificity of 94.2% against blinded expert consensus.

Across large, externally validated systems, within‐study AUC variation across cohorts was observed but its interpretation is limited by the non‐comparable nature of the cohorts involved. Tromp et al. [13] reported AUCs of 0.96, 0.91, and 0.91 for E/e′ ≥13 across Asia, Canada, and Taiwan respectively. The higher value in the smaller Asian cohort may reflect proximity to training data, though this cannot be established from a single study's validation results. The consistency of the two larger independent cohorts at 0.91 is noted, though two cohorts from a single study are insufficient to establish a stable real‐world estimate. Independent replication across separate research groups would be required to confirm this. Cai et al. [23] reported an internal diastolic parameter AUC of 0.99. EchoNet‐Dynamic and CAMUS were used for technical pipeline validation of segmentation and LVEF components only and do not represent external clinical validation of diastolic targets. Diastolic performance estimates for Cai et al. are therefore derived from internal testing only. Across the guideline‐based stratum, AUCs ranged from 0.88 to 0.992 depending on evidence type and validation approach. Because these reflect non‐equivalent tasks — parameter‐level discrimination, full guideline‐grade agreement, and surrogate‐label classification — no single summary statistic is reported. Table 4 presents AUCs stratified by evidence type and validation approach.

**TABLE 4:** Arm‐level summary of diagnostic performance by evidence stratum.

Study (year)	Target condition	Reference standard	Index‐test output	Validation cohort, n	AUC (95% CI)	Evidence stratum / contribution to synthesis
**Stratum 1: Invasive hemodynamic reference studies**						
Pandey et al. (2021) [19] Primary invasive arm	Elevated LV filling pressure	Invasive PCWP or LV pre‐A pressure >15 mmHg (RHC or LHC)	DeepNN probability score	*n* = 84 (full cohort incl. guideline‐indeterminate)	0.883 (CI NR)	Stratum 1 – Primary estimate (included in reported AUC range)
Pandey et al. (2021) [19] Guideline‐classifiable subgroup	Elevated LV filling pressure	Same as above	DeepNN probability score	Guideline‐classifiable subset only (n not separately reported)	*0.894 (CI NR)*	Stratum 1 – Secondary subgroup analysis (reported descriptively only)
Xu et al. (2024) [21] Primary target: LVEDP	Elevated LVEDP	Invasive LVEDP >20 mmHg (LHC)	Logistic regression (best performing model)	≈46 per iteration; 20 repeated 9:1 splits	0.761 (0.725–0.796)	Stratum 1 – Primary estimate (included in reported AUC range)
Xu et al. (2024) [21] Secondary target: tau	Impaired relaxation	Invasive tau >45 ms (LHC)	Gradient boosting (best for tau)	≈46 per iteration; 20 repeated 9:1 splits	*0.832 (0.700–0.964)*	Stratum 1 – Secondary physiological target (reported descriptively only)
Gruca et al. (2023) [16]	Elevated LVEDP	Invasive LVEDP ≥15 mmHg (LHC)	LASi (PLS regression)	*n* = 194 (internal hold‐out)	*No AUC reported Accuracy 0.79; Sn 0.80; Sp 0.77*	Stratum 1 – No ROC analysis reported (excluded from AUC range)
Khan et al. (2026) [25]	Elevated LV filling pressure	Invasive PAWP or LV pre‐A >15 mmHg, or LVEDP >16 mmHg	Logistic regression (best performing classifier)	*n* = 250 aggregate out‐of‐fold test predictions (nested 5‐fold CV)	0.87 (CI NR)	Stratum 1 – Primary estimate (included in reported AUC range)
Nakamura et al. (2026) [26] Model 1 (5‐variable)	Elevated LV filling pressure	Invasive PAWP ≥18 mmHg (RHC)	XGBoost classifier (Model 1: E/A, e′, E/e′, LAVi, TRV)	*n* = 335 (independent external validation cohort)	0.81 (0.75–0.87)	Stratum 1 – External validation (reported descriptively)
Nakamura et al. (2026) [26] Model 2 (9‐variable)	Elevated LV filling pressure	Invasive PAWP ≥18 mmHg (RHC)	XGBoost classifier (Model 2: Model 1 variables + IVC, LVEDD, LAD, LVEF)	*n* = 335 (independent external validation cohort)	0.84 (0.79–0.89)	Stratum 1 – Primary external validation estimate (included in reported AUC range)
**Stratum 2: Non‐invasive reference studies: guideline‐, expert‐, or surrogate‐label reference standards**						
Qu et al. (2023) [14]	Diastolic dysfunction versus normal	Clinician‐assigned labels; guideline basis not specified	FFANet binary classifier	≈57 (internal 8:2 split test set; corrected from total *n* = 286)	0.922 (CI NR)	Stratum 2 – Primary estimate (included in reported AUC range)
Chen et al. (2023) [22] External prospective test	LVDD detection and grading	Two‐expert ASE/EACVI 2016 classification	Multiview AI‐assisted workflow	*n* = 388 (external prospective test set)	*Accuracy 0.90 (detection), 0.92 (grading); AUC NR*	Stratum 2 – External prospective validation (accuracy reported; no ROC analysis)
Gottlieb et al. [18] (2024)	Diastolic dysfunction	Blinded two‐expert consensus (pre‐specified criteria)	Vendor embedded AI (Mindray Auto‐DFR)	*n* = 209 (prospective, 220 enrolled; 11 excluded)	*Sn 91.9% (85.6–96.0); Sp 94.2% (87.0–98.1); AUC NR*	Stratum 2 – Prospective clinical evaluation (accuracy only; no ROC analysis)
Karužas et al. (2025) [20]	LVDF grading (grades 0–3)	Expert manual ASE/EACVI assessment (single reviewer)	Commercial automated CNN (Ligence Heart)	*n* = 302 overall; n = 296 evaluable for grade‐level performance	*Grade‐specific precision, recall, F1 reported; AUC NR*	Stratum 2 – Prospective clinical evaluation (grade‐specific metrics only)
Salem Omar et al. (2018) [17]	Elevated LVFP surrogate (E/e′ ≥13)	Non‐invasive surrogate: E/e′ ≥13	Ensemble (RF + ANN + SVM; majority vote) on STE features	Training *n* = 130; held‐out test *n* = 44	*14 features: CV‐AUC 0.847/test 0.868* *11 features: CV‐AUC 0.853/test 0.881 (all CI NR)*	Stratum 2 – Surrogate‐label model (included in surrogate‐label synthesis only); exploratory invasive substudy (*n* = 44) reported descriptively (no invasive AUC available).
Jiang et al. (2022) [24]	Abnormal LVDF and elevated filling pressure (continuous score)	ASE/EACVI 2016 guideline‐based labels (non‐invasive; indeterminate excluded)	Tabular ML ensemble + R‐DNN continuous score	*n* = 5,798 (internal 20% validation split)	*AUC 0.99 (abnormal DF); 0.99 (elevated FP by score threshold) (CI NR; internal validation only)*	Stratum 2 – Primary estimate (included in reported AUC range); internal validation after exclusion of indeterminate cases
Cai et al. (2024) [23] Final LVDF grading	Final LVDF grade (guideline‐based)	Expert ASE/EACVI 2016 grading	MMnet hybrid DL + ML pipeline (probabilistic grading)	*n* = 212 evaluable for grading (internal test *n* = 239 overall)	*Hard‐rule accuracy 0.88; probabilistic accuracy up to 0.98 AUC NR for final grading*	Stratum 2 – Final LVDF grading (accuracy only; parameter AUCs reported separately in Stratum 3)
**Stratum 3: Clinical parameter‐replication studies**						
Tromp et al. (2022) [13] Internal test	E/e′ ≥13 and lateral e′ <10 cm/s	Expert manual measurements (sonographer)	End‐to‐end automated DL workflow	*n* = 406 (internal held‐out test set)	E/e′ ≥13: AUC 0.96 Lateral e′ <10: AUC 0.95 (CI NR)	Stratum 3 – Internal parameter replication
Tromp et al. (2022) [13] Canada external	E/e′ ≥13 and lateral e′ <10 cm/s	Expert manual measurements	Same DL workflow; no retuning	*n* = 1,029 (external validation)	E/e′ ≥13: AUC 0.91 Lateral e′ <10: AUC 0.88 (CI NR)	Stratum 3 – External parameter replication
Tromp et al. (2022) [13] Taiwan external	E/e′ ≥13 and lateral e′ <10 cm/s	Expert manual measurements	Same DL workflow; no retuning	*n* = 31,241 (largest external cohort)	E/e′ ≥13: AUC 0.91 Lateral e′ <10: AUC 0.94 (CI NR)	Stratum 3 – External parameter replication
Park et al. (2024) [15] Parameter detection	Individual guideline diastolic thresholds	Expert manual measurements (TOMTEC independent observer)	Automated pipeline with LA strain	*n* = 200 (external clinical validation)	Septal e′ <7: AUC 0.961 E/e′ >15: AUC 0.966 TR Vmax >2.8: AUC 0.967 LAVi >34: AUC 0.977 (CI NR)	*Stratum 3 –* External parameter replication. *Park* et al. *also contributes to Stratum 2 for LVDF grading agreement*
Cai et al. (2024) [23] Parameter threshold replication	Individual guideline diastolic parameter thresholds	Expert manual measurements (internal test set only)	MMnet automated measurement pipeline	*n* = 239 internal test; parameter‐specific available‐case denominators	E/e′ >14: AUC 0.992 (0.977–0.997) Septal e′ <7: AUC 0.990 (0.971–0.998) Lateral e′ <10: AUC 0.995 (0.968–0.998) TRmax >280: AUC 0.977 (0.956–0.991)	Stratum 3 – Internal parameter replication only. Technical validation datasets (EchoNet‐Dynamic and CAMUS) evaluated segmentation and LVEF components and are excluded from the clinical diastolic‐performance synthesis. Cai et al. also contributes to Stratum 2 through the separate LVDF grading analysis.

*Note*: Diagnostic performance estimates are presented by study arm, including the target condition, reference standard, index‐test output, validation cohort, and AUC (95% CI), where available. Studies are grouped into three predefined evidence strata according to the clinical task evaluated. Where multiple analyses were reported within a study, each arm is presented separately and its contribution to the narrative synthesis is specified. No pooled estimate was calculated because the strata represent non‐equivalent clinical tasks.

Abbreviations: ANN, artificial neural network; ASE, American society of echocardiography; AUC, area under the receiver operating characteristic curve; CI, confidence interval; CNN, convolutional neural network; CV, cross‐validation; DL, deep learning; EACVI, European association of cardiovascular imaging; FFANet, feature fusion attention network; LAD, left atrial dimension; LASi, left atrial strain index; LAVi, left atrial volume index; LHC, left‐heart catheterization; LVDF, left ventricular diastolic function; LVEDD, left ventricular end‐diastolic diameter; LVEDP, left ventricular end‐diastolic pressure; LVEF, left ventricular ejection fraction; LVFP, left ventricular filling pressure; MMnet, multimodal neural network; NR, not reported; PAWP, pulmonary artery wedge pressure; PCWP, pulmonary capillary wedge pressure; PLS, partial least squares; R‐DNN, regression deep neural network; RF, random forest; RHC, right‐heart catheterization; ROC, receiver operating characteristic; Sn, sensitivity; Sp, specificity; STE, speckle‐tracking echocardiography; SVM, support vector machine; TRV, tricuspid regurgitation velocity; XGBoost, extreme gradient boosting.

### 3.6 Parameter‐Level Replication of Diastolic Indices

Three studies evaluated AI measurement replication of individual guideline‐based diastolic parameters, reporting agreement with expert manual measurements for prespecified clinical thresholds (Tromp et al. [13]; Cai et al. [23]; Park et al. [15]). These studies compared automated AI‐derived measurements with manual expert measurements of the same echocardiographic parameters; they did not evaluate diagnostic accuracy against an independent clinical reference standard, and findings are described using measurement agreement and replication terminology throughout. Reporting was most complete in large, fully automated pipelines, while smaller or single‐center studies variably reported operating characteristics and confidence intervals. Given that thresholds, acquisition pipelines, and validation cohorts differed across studies, direct comparisons between agreement values across studies should not be drawn, and findings are described within each study only.

For E/e′ ratio, automated measurement replication was consistently strong across multicohort evaluations. Tromp et al. [13] reported AUCs of 0.96, 0.91, and 0.91 for agreement with expert‐measured E/e′ ≥13 in Asian, Canadian, and Taiwanese cohorts, respectively. Cai et al. [23] reported an AUC of 0.992 for automated replication of E/e′ >14 using an end‐to‐end automated pipeline, while Park et al. [15] reported an AUC of 0.966 for automated replication of E/e′ >15 in an external validation cohort. These values were obtained at different thresholds, using different pipelines, and in non‐comparable populations, and should be interpreted as study‐level observations rather than directly comparable estimates.

Tissue Doppler velocities also showed high measurement agreement. Lateral e′ <10 cm/s yielded AUCs ranging from 0.88 to 0.95 in Tromp et al., and 0.995 in Cai et al. [23]. Septal e′ <7 cm/s showed similarly strong automated replication within the studies that reported it, with AUCs at or above 0.95 in Cai et al. and Park et al. [15, 23]. Tricuspid regurgitation velocity and left atrial volume index also showed high measurement agreement AUCs in Park et al. (0.967 and 0.977 respectively) [15]. Whether this pattern reflects any genuine difference in automated replication performance across parameter types cannot be determined from this evidence base, as thresholds, populations, and pipelines were non‐comparable across studies, and these parameters were not evaluated alongside each other in independent external cohorts. Within‐study parameter variation was small in both Park et al. [15] (range 0.961–0.977) and Cai et al. [23] (range 0.977–0.995), suggesting consistent automated pipeline measurement replication across parameter types within those studies.

Because parameter definitions, clinical thresholds, uncertainty reporting, and validation cohorts were not harmonized, and several parameters were reported by only one or two studies, quantitative pooling was not performed. Measurement replication results were therefore synthesized descriptively, with emphasis on consistency across large, externally validated automated pipelines. Measurement agreement AUCs are summarized in Table S2.

### 3.7 Indeterminate Classifications, Feasibility, and Failure Rates

Across studies, AI‐based approaches consistently reduced indeterminate classifications compared with guideline‐based diastolic assessment. In invasive‐reference cohorts, AI systems generated determinate outputs for nearly all patients, whereas guideline algorithms frequently yielded indeterminate results. In Pandey et al. [19], guideline‐based assessment was indeterminate in 17.9% of patients undergoing invasive hemodynamic evaluation and in 42.5% of their cohort from the TOPCAT trial, while the AI phenogrouping model produced determinate classifications for all patients. Similarly, Gruca et al. [16] reported guideline indeterminacy in 19.1% of cases, whereas their left atrial strain–based index generated determinate outputs and retained performance within the guideline‐indeterminate subgroup. More recent studies reinforced this finding: Khan et al. [25] generated determinate classifications for all patients, compared with unclassified rates of 13% and 3% using the 2016 ASE/EACVI and 2022 EACVI algorithms, respectively. Nakamura et al. [26] also classified all patients despite 42.7% being indeterminate by the guideline algorithm, with Model 2 retaining an AUROC of 0.84 in this subgroup.

In guideline‐based studies, fully automated multiview pipelines also improved feasibility. Chen et al. showed that automated workflows could assign diastolic grades despite missing parameters, with single‐view fallback models preserving performance, while Park et al. reported high measurement yield and strong concordance between AI‐derived and manual grades [15, 22]. Feasibility varied by setting: multiview laboratory‐based systems achieved the highest yields, whereas emergency department and point‐of‐care deployments had higher exclusion or failure rates. In the emergency department study by Gottlieb et al. [18], 5.0% of examinations were excluded due to inadequate image quality. Commercial automation showed high success rates for transmitral inflow and tissue Doppler parameters, with lower measurement yield for left atrial volume index (73%) [20].

### 3.8 Risk of Bias and Applicability

Risk‐of‐bias patterns varied across QUADAS‐3 domains (Supplementary Material S3/S4). Externally validated multiview pipelines generally demonstrated lower risk in index‐test and flow/timing domains, particularly when evaluated using fixed automated workflows without study‐side tuning. However, concerns related to patient selection, applicability, exclusion criteria, and reference standards remained present across several studies. Patient‐selection concerns were common and were primarily driven by retrospective, non‐consecutive cohorts and extensive exclusions, particularly in Cai et al., Jiang et al., and Qu et al. [14, 23, 24]. In contrast, Tromp et al. and Xu et al. were judged at low risk for patient selection [13, 21]. Index test bias was generally low for fixed, fully automated pipelines evaluated without study‐side tuning, but was higher or unclear where models were trained to reproduce guideline labels derived from the same inputs (Jiang et al. [24]), thresholds were optimized on training data (Chen et al. [22]), or model architecture and calibration were incompletely reported (Qu et al. [14]; Salem Omar et al. [17]).

Reference‐standard risk of bias was generally lowest in studies using invasive hemodynamics, including Pandey et al. [19], Gruca et al. [16], Xu et al. [21], Khan et al. [25], and Nakamura et al. [26]. In non‐invasive reference studies, the principal concerns related less to the internal application of the reference standard itself and more to applicability, because guideline‐derived, expert‐derived, or surrogate labels do not directly establish elevated invasive filling pressure. Higher risk‐of‐bias judgements were assigned where reference definitions were poorly specified, surrogate labels were used, indeterminate cases were excluded, or potential circularity existed between model inputs and reference labels. Flow and timing were generally appropriate, although incomplete reporting limited assessment in some studies.

## 4 Discussion

Accurate assessment of left ventricular diastolic function and filling pressures remains a major challenge in contemporary heart‐failure care, particularly in HFpEF, where echocardiography is central to diagnosis but guideline‐based algorithms are frequently indeterminate, variably reproducible, and difficult to operationalize in routine practice [2, 4, 9]. These limitations contribute to delayed or missed diagnoses, heterogeneous clinical decision‐making, and substantial inter‐operator variability [6, 7]. AI has therefore emerged as a potential strategy to standardize measurement, integrate multiparametric echocardiographic signals, and improve the consistency of non‐invasive diastolic assessment [10, 11, 12].

### 4.1 Diagnostic Performance Versus Invasive Hemodynamic Reference

Evidence comparing AI‐derived echocardiographic assessments with invasive hemodynamics remains heterogeneous but has expanded considerably. Among studies reporting ROC‐based performance, AUROCs ranged from 0.761 to 0.883: Pandey et al. achieved an AUROC of 0.883, Xu et al. 0.761, Khan et al. 0.87, and Nakamura et al. 0.81–0.84, while Gruca et al. reported accuracy rather than AUC [16, 19, 21, 25, 26]. Given the differences in invasive targets, thresholds, populations, and validation approaches across these studies, no summary figure is reported. Performance gains over guideline‐based grading were most apparent in cohorts containing guideline‐indeterminate cases, where AI systems generated determinate outputs and maintained discrimination against invasive hemodynamics.

Among the earlier studies, Pandey et al. [19] provided a clinically informative comparison by demonstrating superior discrimination relative to guideline‐based grading in a catheterization cohort, driven largely by resolution of indeterminate classifications. Gruca et al. [16] highlighted the value of alternative signal representations, showing that use of the full left atrial strain time curve improved accuracy over both peak strain and guideline‐based assessment. Xu et al. [21] extended this concept by integrating echocardiographic variables with NT‐proBNP and clinical data, demonstrating that tabular machine‐learning approaches can capture complementary hemodynamic information not reflected in individual echocardiographic parameters. Khan et al. [25] subsequently demonstrated similar discrimination across a larger multinational, multicenter cohort using nested cross‐validation, with all evaluated machine‐learning models generating determinate classifications. Nakamura et al. [26] further strengthened the evidence base by demonstrating that explainable XGBoost models retained good discrimination when tested unchanged in a genuinely independent external hospital cohort.

The within‐study subgroup analyses are arguably more clinically informative than the overall AUCs. In Pandey et al. [19], the AI model outperformed guideline grading in the full cohort primarily because it generated determinate classifications for cases where guideline assessment failed — performance in guideline‐classifiable cases was similar between the two approaches (AUROC 0.89 vs. 0.83; *p* = 0.32). Similarly, Nakamura et al. showed that Model 2 retained an AUROC of 0.84 in guideline‐indeterminate cases, while both models outperformed the guideline algorithm among classifiable patients [26]. In Xu et al. [21], the divergence between LVEDP prediction (AUROC 0.761) and tau prediction (AUROC 0.832) in the same cohort illustrates that different invasive hemodynamic targets are not interchangeable, and that model performance is intrinsically tied to the physiological construct being predicted. Collectively, these findings suggest that a key clinical role for AI may be the resolution of diagnostically challenging cases, rather than wholesale replacement of guideline assessment.

However, important limitations remain. The invasive studies used heterogeneous hemodynamic targets and thresholds, and differences in populations, catheterization methods, timing of echocardiography relative to invasive assessment, and modelling approaches limit direct comparison. Nakamura et al. [26] represents the most methodologically robust invasive‐reference study identified in this review, providing independent external validation against PAWP in a distinct hospital cohort and demonstrating maintained discrimination in guideline‐indeterminate patients. Nevertheless, no study has yet demonstrated clinical impact within a prospective AI‐guided diagnostic pathway. The small exploratory invasive substudy reported by Salem Omar et al. [17], derived from a model trained on non‐invasive surrogate labels, further illustrates the variable strength of invasive validation across the evidence base.

### 4.2 Diagnostic Performance Versus Guideline‐Based Diastolic Function

The largest body of evidence addressed automation or replication of guideline‐based diastolic assessment. Fully automated multiview pipelines with external or multicohort validation demonstrated the most consistent and generalizable performance. Tromp et al. provided particularly strong evidence, validating an end‐to‐end workflow across multiple continents and reporting consistently high discrimination for key diastolic indices with lower measurement variance than expert sonographers [13]. Similarly, Cai et al. and Park et al. showed that automated systems could reproduce guideline‐based grading with high accuracy and strong agreement across core parameters, supporting scalable and standardized assessment [15, 23]. Importantly, several of these multiview studies primarily evaluated automated parameter extraction and measurement replication against expert annotations rather than direct diagnostic validation against independent hemodynamic outcomes.

The modest attenuation in AUC observed between the smaller Asian cohort (0.96) and the two larger independent cohorts in Tromp et al. (0.91 each) is consistent with expected internal validation optimism and may represent a more conservative estimate of real‐world performance, though this interpretation is based on a single study's validation results and would require independent replication to confirm [13]. The consistency of the two larger external cohorts at 0.91 is noted as an observation within this study. Cai et al. reported an internal diastolic parameter AUC of 0.99 [23]. However, EchoNet‐Dynamic and CAMUS were used to validate technical pipeline components, specifically segmentation and LVEF measurement, rather than to externally validate diastolic dysfunction classification. The results from these datasets therefore do not constitute evidence of an internal‐to‐external performance gradient for diastolic targets and are not included in the diastolic‐performance synthesis. Diastolic performance estimates for Cai et al. [23] are derived from internal testing only.

Prospective evaluation by Chen et al. [22] further demonstrated that automated multiview workflows can maintain diagnostic performance even when guideline parameters are incomplete, through the use of fallback models. In contrast, evidence from tabular, commercial, morphology‐only, and point‐of‐care approaches was more heterogeneous. While Jiang et al. reported near‐perfect internal discrimination, this performance was achieved in a fully classifiable cohort with exclusion of guideline‐indeterminate cases and training on labels derived from the same inputs, raising concerns regarding circularity and generalizability [24]. Single‐view morphology‐based classification and emergency department deployment showed feasibility but were limited by lack of external validation, reliance on accuracy‐based metrics, or image‐quality exclusions [14, 18].

Across studies reporting ROC‐based performance against guideline‐based or expert reference standards, AUCs ranged from 0.88 (Salem Omar et al., surrogate‐label cross‐validation) to 0.995 (Cai et al., parameter‐level internal validation) [17, 23]. Because AUCs in this stratum reflect non‐equivalent tasks, spanning internal and external validation cohorts of widely varying size, no single summary statistic is reported. The highest AUCs within their respective validation contexts came from large, externally validated multiview pipelines (Tromp: 0.91–0.96 across cohorts; Park: 0.966 external validation; Cai: 0.992 internal), though direct comparison across these studies is not appropriate given differences in tasks, thresholds, and populations [13, 15, 23]. Overall, agreement with guideline‐based classification appears most robust when AI systems automate both measurement and integration across multiple views and modalities, rather than reproducing guideline logic from pre‐derived variables alone.

### 4.3 Parameter‐Level Replication of Diastolic Indices

Parameter‐level analyses demonstrated consistently high discrimination for individual diastolic thresholds, particularly in large, fully automated pipelines. Across multiple cohorts and acquisition environments, AI systems reliably classified E/e′, tissue Doppler velocities, and related parameters, often with lower variance than manual measurement [13, 15, 23]. However, reporting was uneven, with variable thresholds, incomplete confidence intervals, and selective parameter inclusion. Because thresholds, populations, and pipelines differed across studies, parameter‐level AUC values should not be interpreted as directly comparable between studies. These findings therefore support technical robustness of automated extraction rather than interchangeable diagnostic endpoints and do not justify pooled quantitative synthesis.

### 4.4 Indeterminate Classifications, Feasibility, and Failure Rates

Reduction of indeterminate classifications emerged as a consistent advantage of AI‐based approaches. In invasive‐reference studies, AI systems generated determinate outputs for nearly all patients, including those classified as indeterminate by guideline algorithms [16, 19]. Similar improvements were seen in guideline‐based studies, where multiview automated systems could assign grades despite missing parameters [15, 22].

Measurement feasibility varied by setting and pipeline. Fully automated laboratory‐based systems achieved the highest yields, whereas emergency department and point‐of‐care deployments showed higher rates of exclusion due to image quality [18]. Commercial automation showed strong performance for core Doppler indices but reduced yield for left atrial volume. These findings highlight the influence of acquisition environment and signal availability on real‐world performance.

### 4.5 Certainty of Evidence

Certainty was judged narratively by evidence type to prevent readers from interpreting the three strata as clinically equivalent, without application of a formal grading scheme.

For invasive hemodynamic prediction, five studies provided direct invasive validation, including a multinational multicenter study using nested cross‐validation and a multicenter study with independent external validation. Confidence in the generalizability of the reported discrimination estimates is nonetheless limited by heterogeneous invasive targets and thresholds, differences in study populations and validation approaches, and the absence of prospective interventional clinical‐impact studies, and limited prospective multicenter validation against invasive hemodynamics.

For guideline‐label agreement, external validation is available for multiview pipelines, but reference standards are non‐independent, indeterminate cases are frequently excluded, and populations are highly selected. Confidence in how well these results reflect unselected clinical practice is therefore limited.

For parameter‐level replication, large externally validated pipelines provide consistent evidence of automated measurement accuracy across multiple cohorts. However, the clinical task is measurement automation rather than diagnostic accuracy for elevated filling pressure, which limits the directness of inference for clinical decision‐making.

### 4.6 Limitations

This review has several limitations. The evidence base remains heterogeneous, precluding quantitative meta‐analysis for most outcomes. Many included studies were retrospective and single‐center, with non‐consecutive sampling and extensive exclusions, contributing to patient‐selection and applicability concerns across QUADAS‐3 domains. Reference standards varied widely, particularly among invasive studies, and although direct hemodynamic validation has been strengthened by recent multicenter and external validation, invasive targets and thresholds remain non‐harmonized across cohorts.

A particularly important applicability concern is the near‐universal exclusion of patients with atrial fibrillation, significant mitral valve disease, pacing rhythms, conduction abnormalities, and poor image quality across included studies. These conditions are common in real‐world diastology practice and represent precisely the populations in whom guideline‐based assessment is most frequently indeterminate and for whom AI tools would theoretically offer the greatest clinical benefit. Current evidence therefore cannot be assumed to generalize to unselected clinical populations, and future validation studies should prospectively include or separately analyze these subgroups.

Methodological quality also varied substantially. Higher risk of bias was observed in studies relying on proxy or guideline‐derived labels, excluding guideline‐indeterminate cases, or using morphology‐only or poorly described model architectures. Inconsistent reporting of calibration, uncertainty, and failure modes further limits interpretability and generalizability.

While several included studies incorporated prospective cohorts or prospective external validation, including emergency department deployment, prospective commercial software evaluation, and multicenter parameter replication, few studies evaluated downstream clinical impact or integration into decision‐making pathways. No study evaluated AI‐based diastolic assessment within a prospective interventional clinical pathway, and prospective multicenter validation against invasive hemodynamics remains limited. Accordingly, reported discrimination metrics should be interpreted as descriptive rather than definitive estimates of clinical performance.

Funding and commercial involvement varied across included studies and are summarized in Table S9. Several studies were supported by governmental or academic funding bodies, while some studies reported commercial involvement, including author employment, equity, patents, consultancy, honoraria, grant support, or evaluation of vendor‐integrated/proprietary AI software. These disclosed relationships represent potential sources of bias and should be considered when interpreting reported diagnostic performance. Independent external validation remains essential before widespread clinical adoption of proprietary AI systems.

A further limitation is the near‐universal absence of calibration reporting. No included study reported calibration metrics such as Brier scores, calibration curves, or Hosmer–Lemeshow statistics. Calibration reflects the agreement between predicted probabilities and observed outcomes and is a distinct property from discrimination. A model with a high AUC may nonetheless assign systematically miscalibrated probabilities, which is of particular relevance where probabilistic outputs are used to resolve guideline‐indeterminate cases. This gap should be a mandatory component of future reporting standards.

### 4.7 Near‐Scope Evidence and Positioning Against Prior Reviews

This review focuses specifically on supervised AI systems evaluated for diagnostic accuracy against a prespecified reference standard for diastolic dysfunction or elevated filling pressure. Two categories of adjacent evidence were intentionally excluded from the formal synthesis. Unsupervised machine‐learning approaches for diastolic phenotyping, including topological data analysis and clustering methods, identify data‐driven phenogroups whose hemodynamic correlates are validated post hoc rather than prospectively against a prespecified reference standard [28, 29]. These represent a complementary parallel literature that is not amenable to the same diagnostic accuracy framework applied here. AI tools developed for HFpEF detection as a clinical syndrome were similarly excluded where the index test targeted the diagnosis of HFpEF rather than diastolic function or filling pressure per se, as these involve distinct reference standards and outcome definitions [30].

### 4.8 Implications for Clinical Implementation and Future Research

For AI‐based diastolic assessment to transition into routine clinical use, several requirements remain unmet. Further prospective multicenter validation against harmonized invasive hemodynamic reference standards is needed, with particular emphasis on prospective clinical pathways and diverse external populations, alongside evaluation of calibration, robustness across acquisition environments and cardiac rhythms, and downstream clinical and cost‐effectiveness outcomes. Regulatory pathways require prospectively defined intended use and predefined performance thresholds, and workflow integration across heterogeneous institutional environments remains largely unaddressed by existing studies. Questions of liability when AI outputs contribute to clinical decisions, and the risk of automation bias attenuating real‐world benefit, are further considerations that technical validation alone cannot resolve. The chronological trajectory of included studies nonetheless offers some optimism, as earlier work (Salem Omar et al. [17], Jiang et al. [24]) was characterized by predominantly single‐center and internally validated approaches, whereas later studies (Chen et al. [22]; Cai et al. [23]; Park et al. [15]) increasingly employed end‐to‐end multiview deep learning, multicenter validation, and stronger evaluation designs. Most recently, Nakamura et al. [26] provided independent external validation against invasive PAWP in a distinct hospital cohort, representing an important progression towards clinically generalizable AI‐based filling‐pressure assessment.

## 5 Conclusion

AI–based echocardiographic assessment of diastolic function and filling pressures shows substantial promise for improving non‐invasive evaluation of heart failure, particularly HFpEF. Against invasive hemodynamic references, reported AUCs ranged from 0.761 to 0.883 across heterogeneous targets and validation approaches. The invasive evidence base has strengthened with recent multicenter studies, including independent external validation, although differences in hemodynamic definitions and study design preclude a single summary estimate. Studies evaluating agreement with guideline‐ or expert‐derived classifications and automated replication of individual diastolic parameters generally reported high discrimination, but these represent non‐equivalent clinical tasks and should not be interpreted as direct evidence of diagnostic accuracy for elevated filling pressure. The evidence base remains heterogeneous, with variable reference standards, limited calibration reporting, and an absence of prospective clinical‐impact studies, and limited prospective multicenter validation against invasive hemodynamics. Future work should prioritize harmonized invasive validation, calibration, evaluation in representative clinical populations, and demonstration of clinical and economic impact to support routine adoption.

## Funding

This research received no external funding.

## Disclosure

No AI tools were used in the generation of the manuscript text or figures, including the graphical abstract.

## Ethics Statement

This study is a systematic review of previously published data. No primary data were collected and no human participants or animals were involved directly. Ethical approval and patient consent were therefore not required. The review protocol was prospectively registered with PROSPERO (CRD420251140771).

## Conflicts of Interest

The authors report no conflicts of interest.

## Supporting information




**Supporting Information**: echo70630‐sup‐0001‐SuppMat.docx

## Data Availability

No new data were generated or analyzed in this study. All data presented are derived from previously published studies cited in the reference list.
