# Supplementary material for: Artificial Intelligence–Based Echocardiographic Assessment of Diastolic Dysfunction: A Systematic Review
Source: Echocardiography. 2026 Sep 23;43(9):e70630. doi: 10.1111/echo.70630 (PMC13598620; doi:10.1111/echo.70630)
Supplement: Supplementary file 1 [file ECHO-43-e70630-s001.docx]

# Supplementary material S1 – Systematic search strategy.

Searches were rerun on 7 July 2026. Platforms searched were PubMed/MEDLINE, Embase, Web of Science Core Collection, and IEEE Xplore. Searches were run from database inception to 7 July 2026, with no language restrictions.

## PubMed (MEDLINE)

( "Echocardiography"[Mesh] OR echocardiograph*[tiab] OR "transthoracic echocardiography"[tiab]

OR TTE[tiab] OR (cardiac[tiab] AND ultrasound[tiab]) OR "point-of-care ultrasound"[tiab]

OR POCUS[tiab] )

AND

( "Diastole"[Mesh] OR "Ventricular Dysfunction, Left"[Mesh] OR "Heart Failure"[Mesh]

OR diastol*[tiab] OR "filling pressure*"[tiab] OR "left ventricular filling"[tiab]

OR PCWP[tiab] OR LVEDP[tiab]

OR "E/e'"[tiab] OR "E/e'"[tiab] OR "E/E'"[tiab] OR "E/Ea"[tiab] OR "E over e"[tiab]

OR "E to e"[tiab] OR "tissue doppler"[tiab] OR "Doppler Tissue Imaging"[tiab]

OR "Echocardiography, Doppler, Tissue"[Mesh] OR "left atrial strain"[tiab]

OR "diastolic dysfunction"[tiab] OR "HFpEF"[tiab] )

AND

( "Artificial Intelligence"[Mesh] OR "Machine Learning"[Mesh] OR "Deep Learning"[Mesh]

OR "Neural Networks, Computer"[Mesh] OR "automation"[Mesh]

OR "artificial intelligence"[tiab] OR "machine learning"[tiab] OR "deep learning"[tiab]

OR "neural network"[tiab] OR "neural networks"[tiab] OR convolutional[tiab]

OR transformer*[tiab] OR "support vector"[tiab] OR "random forest"[tiab]

OR "gradient boosting"[tiab] OR "automated"[tiab] OR "automation"[tiab]

OR "computer-aided"[tiab] OR "algorithm"[tiab] OR "natural language processing"[tiab]

OR "foundation model"[tiab] OR "large language model"[tiab] )

**Embase**

## ( "echocardiography"/exp

## OR "transthoracic echocardiography":ti,ab

## OR echocardiograph*:ti,ab

## OR TTE:ti,ab

## OR "point-of-care ultrasound":ti,ab

## OR POCUS:ti,ab

## OR (cardiac:ti,ab AND ultrasound:ti,ab) )

## AND

## ( "diastolic dysfunction"/exp

## OR "left ventricular filling pressure"/exp

## OR (diastol* NEAR/3 function):ti,ab

## OR (diastol* NEAR/3 dysfunction):ti,ab

## OR (filling NEAR/2 pressure):ti,ab

## OR "left ventricular filling":ti,ab

## OR pcwp:ti,ab

## OR lvedp:ti,ab

## OR "tissue doppler":ti,ab

## OR "doppler tissue imaging":ti,ab

## OR "left atrial strain":ti,ab

## OR "E/e'":ti,ab

## OR HFpEF:ti,ab )

## AND

## ( "artificial intelligence"/exp

## OR "machine learning"/exp

## OR "deep learning"/exp

## OR "neural network"/exp

## OR "computer assisted diagnosis"/exp

## OR "machine learning":ti,ab

## OR "deep learning":ti,ab

## OR "artificial intelligence":ti,ab

## OR neural*:ti,ab

## OR convolutional:ti,ab

## OR transformer*:ti,ab

## OR "support vector":ti,ab

## OR "random forest":ti,ab

## OR "gradient boosting":ti,ab

## OR "automated":ti,ab

## OR "computer-aided":ti,ab

## OR "algorithm":ti,ab

## OR "foundation model":ti,ab )

## Web of Science

TS=("artificial intelligence" OR "machine learning" OR "deep learning"

OR "neural network*" OR transformer* OR convolutional

OR "support vector" OR "random forest" OR "gradient boosting"

OR "automated" OR "computer-aided" OR "algorithm*"

OR "foundation model")

AND

TS=(echocardiograph* OR "transthoracic echocardiography" OR TTE

OR "point-of-care ultrasound" OR POCUS

OR (cardiac NEAR/2 ultrasound))

AND

TS=(diastol* OR "filling pressure" OR "left ventricular filling"

OR PCWP OR LVEDP OR "tissue doppler" OR "doppler tissue imaging"

OR "left atrial strain" OR "E/e'" OR HFpEF

OR "diastolic dysfunction")

## IEEE Xplore

( (echocardiograph* OR "transthoracic echocardiography" OR TTE

OR POCUS OR "point-of-care ultrasound"

OR (cardiac NEAR/3 ultrasound) OR (heart NEAR/3 ultrasound)) )

AND

( diastol* OR "filling pressure" OR "left ventricular filling"

OR PCWP OR LVEDP OR "E/e'" OR "tissue doppler"

OR "diastolic dysfunction" OR HFpEF )

AND

( "artificial intelligence" OR "machine learning" OR "deep learning"

OR "neural network" OR convolutional OR transformer

OR "support vector" OR "random forest" OR "gradient boosting"

OR automated OR "computer-aided" OR "foundation model" )

**Supplementary material S2 - Parameter-level discrimination of AI-derived diastolic indices against guideline-based thresholds**

| **Parameter threshold** | **Study arm** | **Setting / acquisition pipeline** | **AUC (95% CI)** | **N (total)** |
| --- | --- | --- | --- | --- |
| Lateral e′ <10 cm/s | Cai 2024 | End-to-end automated measurements from 2D + Doppler | 0.9947 (0.9684–0.9978) | Internal test: 239 |
| Lateral e′ <10 cm/s | Tromp 2022 — Asia (internal test) | Fully automated view classification, segmentation, Doppler | 0.95 (0.88–0.99) | 406 |
| Lateral e′ <10 cm/s | Tromp 2022 — Canada | Same automated pipeline (external site) | 0.88 (0.84–0.92) | 1,029 |
| Lateral e′ <10 cm/s | Tromp 2022 — Taiwan | Same automated pipeline (external site) | 0.94 (0.93–0.95) | 31,241 |
| Septal e′ <7 cm/s | Cai 2024 | End-to-end automated measurements from 2D + Doppler | 0.990 (0.971–0.998) | Internal test: 239 |
| Septal e′ <7 cm/s | Park 2024 (external test) | Automated view selection, Doppler/LA segmentation | 0.961 (CI NR) | 200 |
| TR Vmax >2.8 m/s | Cai 2024 | End-to-end automated measurements from 2D + Doppler | 0.977 (0.956–0.991) | NR- multiple cohorts |
| TR Vmax >2.8 m/s | Park 2024 (external test) | Automated view selection, Doppler/LA segmentation | 0.967 (CI NR) | 200 |
| LAVI >34 mL/m² | Park 2024 (external test) | Automated LA segmentation and LARS tracking | 0.977 (CI NR) | 200 |
| E/e′ ≥13 | Tromp 2022 — Asia (internal test) | Fully automated pipeline | 0.96 (0.92–0.99) | 406 |
| E/e′ ≥13 | Tromp 2022 — Canada | Fully automated pipeline | 0.91 (0.88–0.94) | 1,029 |
| E/e′ ≥13 | Tromp 2022 — Taiwan | Fully automated pipeline | 0.91 (0.89–0.93) | 31,241 |
| E/e′ >14 | Cai 2024 | End-to-end automated measurements | 0.992 (0.977–0.997) | Internal test: 239 |
| E/e′ >15 | Park 2024 (external test) | Automated view selection, Doppler/LA segmentation | 0.966 (CI NR) | 200 |

Abbreviations: AI, artificial intelligence; AUC, area under the receiver operating characteristic curve; CI, confidence interval; E, early diastolic transmitral inflow velocity; A, late diastolic transmitral inflow velocity; e′, early diastolic mitral annular velocity; LAVI, left atrial volume index; LARS, left atrial reservoir strain; TR Vmax, peak tricuspid regurgitation velocity; 2D, two-dimensional; NR, not reported.

Septal and lateral e′ were analysed separately where reported; thresholds reflect study-specific guideline implementation. AUCs represent discrimination of AI-derived measurements against manually derived parameter thresholds.

**Supplementary Methods S3 – QUADAS-3 risk-of-bias and applicability assessment**

Risk of bias and applicability concerns were assessed using the proposed QUADAS-3 framework described by McInnes et al. Two reviewers (CM, HT) independently assessed each study across all domains. Disagreements were resolved by discussion and, where consensus could not be reached, by adjudication from a third reviewer (RD). Domain-level judgements were assigned as **Low risk of bias, High risk of bias, or Unclear risk of bias. "Not applicable" was used only where a domain was not relevant to a study design.** The signalling questions were adapted to reflect AI-specific diagnostic-accuracy studies while preserving the core structure of QUADAS-3.

**Domains and signalling questions**

Domain 1: Patient selection

Was a consecutive or random sample enrolled? Was a case-control design avoided? Were inappropriate exclusions avoided? Could the included patients have introduced bias relative to the target population?

Domain 2: Index test

Were the index test results interpreted without knowledge of the reference standard? If a threshold was used, was it prespecified or selected to maximise performance on the test set? Were model inputs and architecture sufficiently described to permit independent replication? Was the AI system applied in a manner consistent with its intended clinical use?

Domain 3: Reference standard

Is the reference standard likely to correctly classify the target condition? Were reference standard results interpreted without knowledge of the index test? For guideline-based reference standards, was the classification performed by independent reviewers with defined adjudication procedures?

Domain 4: Flow and timing

Was there an appropriate interval between the index test and the reference standard? Did all patients receive the same reference standard? Were all enrolled patients included in the analysis, with withdrawals and exclusions explained?

**AI-specific considerations**

QUADAS-3 does not include AI-specific signalling questions as a separate domain. Where AI-specific concerns were identified — including potential data leakage arising from study-level rather than patient-level train/test splitting, circularity between index test inputs and reference standard labels, incomplete reporting of model calibration, and absence of external validation — these were documented in the comments column and incorporated into the relevant domain judgment. Specifically, data leakage concerns were reflected in the index test and flow and timing domains, label circularity concerns in the reference standard domain, and population selection concerns in the patient selection domain.

**Applicability assessment**

Applicability concerns were assessed separately from risk of bias using the same three domains (patient selection, index test, reference standard) and the same four-category schema. Applicability judgments reflected whether the enrolled population, index test implementation, and reference standard were consistent with the intended clinical use of AI-based diastolic assessment in routine practice.

**Supplementary material S4 – Study-level QUADAS-3 risk-of-bias and applicability assessment**

|  | **Risk of Bias** | | | | **Applicability concerns** | | |  |
| --- | --- | --- | --- | --- | --- | --- | --- | --- |
| **Study** | **Patient selection** | **Index test** | **Reference standard** | **Flow & timing** | **Patient selection** | **Index test** | **Reference standard** | **Comments supporting QUADAS-3 judgement** |
| **Cai 2024** | **High** | **Low** | **Low** | **Low** | High | Low | High | Internal retrospective development. Reference standard was ASE/EACVI grading rather than invasive haemodynamics. EchoNet-Dynamic and CAMUS evaluated segmentation/LVEF components only and were not considered external clinical validation of diastolic targets. |
| **Chen 2023** | **Unclear** | **Low** | **Low** | **Low** | Low | Low | Low | Multicentre retrospective development with independent prospective external validation (n=388). Two-expert ASE/EACVI reference with adjudication. Automated view screening excluded ~10% before testing. |
| **Gottlieb 2024** | **Low** | **Low** | **Low** | **Low** | High | High | Low | Prospective ED cohort using vendor-embedded AI. Consensus expert reference applied independently. Platform-specific implementation may reduce generalisability. |
| **Gruca 2023** | **High** | **Low** | **Low** | **Low** | High | Unclear | Low | Single-centre retrospective catheterisation cohort. Invasive LVEDP reference within 24 hours. Internal validation only. |
| **Jiang 2022** | **High** | **High** | **High** | **High** | High | Low | High | Indeterminate cases removed. Model trained to reproduce guideline labels generated from the same variables, creating substantial circularity. Study-level rather than patient-level split risks leakage. No external validation. |
| **Karužas 2025** | **Low** | **Low** | **Low** | **Low** | High | High | Low | Prospective single-centre evaluation of commercial software. Reference standard was expert grading rather than invasive haemodynamics. Proprietary model limits transparency. |
| **Pandey 2021** | **Low** | **Low** | **Low** | **Low** | High | Unclear | Low | External invasive validation of DeepNN. Labels derived from phenogroups rather than guideline grades. Model classified all patients, including guideline-indeterminate cases. |
| **Park 2024** | **Low** | **Low** | **Low** | **Low** | High | Low | Low | External clinical validation of automated LVDF grading and parameter detection. Reference based on expert guideline grading rather than invasive haemodynamics. |
| **Qu 2023** | **High** | **High** | **High** | **Low** | High | High | High | Internal single-centre 80:20 split only. Clinician labels without explicit guideline or invasive reference. No external validation or calibration. |
| **Salem Omar 2018** | **High** | **High** | **High** | **Unclear** | High | High | High | Model trained using surrogate E/e′ ≥13 labels. Small exploratory invasive substudy (n=44) only. No external validation. |
| **Xu 2024** | **Low** | **Low** | **Low** | **Low** | High | Low | Low | Repeated train–validation–test splits using invasive LVEDP and tau references. Transparent methodology but no external validation. |
| **Tromp 2022** | **Low** | **Low** | **Low** | **Low** | Low | Low | High | Large multicentre development with independent external validation. Outputs replicate expert measurements rather than predict invasive haemodynamics, so reference standard reflects measurement agreement rather than clinical truth. |
| **Khan, 2026** | **Low** | **Low** | **Low** | **Low** | **Low** | **Low** | **Low** | Prospective multicentre cohort (6 centres across 5 countries) undergoing echocardiography within 24 h of invasive catheterisation. Nested 5-fold cross-validation with embedded feature selection and hyperparameter optimisation prevented information leakage. All patients contributed once to aggregate out-of-fold testing. Multiple ML classifiers were compared against ASE/EACVI algorithms using invasive LV filling pressure as the reference standard. No completely independent external validation cohort was included |
| **Nakamura, 2026** | **Low** | **Low** | **Low** | **Low** | **Low** | **Low** | **Low** | Retrospective multicentre development with geographically distinct independent external validation (training n=621; external test n=335). XGBoost models developed using invasive PAWP ≥18 mmHg measured by right-heart catheterisation. External validation performed without model updating and included explainable SHAP analysis.. |

Legend: Low = low risk of bias; High = high risk of bias; Unclear = insufficient reporting.

**Supplementary Table S5. Cohort-level breakdown by study — sample role and size**

| **Study** | **Cohort/ Dataset** | **Role** | **N** |
| --- | --- | --- | --- |
| **Salem Omar (2018)** | | | |
|  | Main cohort — training | Training | 130 |
|  | Main cohort — test (invasive substudy) | Clinical test | 44 |
| **Pandey (2021)** | | | |
|  | Derivation cohort | Training / development | 1,242 |
|  | Haemodynamic validation cohort | External clinical validation | 84 |
|  | Clinical outcomes cohort | External validation | 219 |
|  | TOPCAT trial cohort | External validation | 518 |
|  | RELAX-HF / NEAT-HFpEF pooled cohort | External validation | 346 |
| **Tromp (2022)** | | | |
|  | ATTRaCT — training | Training | 1,145 |
|  | ATTRaCT — internal test | Internal test | 406 |
|  | Canada cohort | External validation | 1,029 |
|  | Taiwan cohort | External validation | 31,241 |
|  | EchoNet-Dynamic | Technical external validation* | 10,030 |
|  | Prospective reproducibility substudy | Prospective validation | 142 |
| **Qu (2023)** | | | |
|  | Classification dataset (8:2 split) | Training + internal test | 286 |
|  | Segmentation dataset | Training | 91 |
| **Chen (2023)** | | | |
|  | View classifier training | Training | 1,304 |
|  | Segmentation training | Training | 2,238 |
|  | Single-view classifier training | Training | 2,150 |
|  | External prospective test | External clinical test | 388 |
| **Gottlieb (2024)** | | | |
|  | Prospective ED cohort | Prospective clinical test | 209 |
| **Park (2024)** | | | |
|  | AI-Hub multicentre development | Training / development | ~30,000 |
|  | External diagnostic validation | External clinical test | 200 |
|  | Mayo Doppler measurement validation | External validation | 100 |
| **Xu (2024)** | | | |
|  | Full cohort (repeated 9:1 splits x20) | Internal validation only | 460 |
| **Cai (2024)** | | | |
|  | Internal training cohort | Training | 862 |
|  | Internal test cohort (Vivid E95) | Internal test | 239 |
|  | External validation (Epiq-7C) | External validation | 154 |
|  | EchoNet-Dynamic | Technical external validation* | 10,030 |
|  | CAMUS | Technical external validation* | 450 |
| **Karuzas (2025)** | | | |
|  | Prospective single-centre cohort | Prospective clinical test | 302 |
| **Jiang (2022)** | | | |
|  | Training split | Training | 23,188 |
|  | Internal validation split | Internal validation | 5,798 |
| **Gruca (2023)** | | | |
|  | Training cohort | Training | 100 |
|  | Hold-out validation cohort | Internal clinical test | 194 |
| **Khan (2026)** | | | |
|  | Nested 5-fold cross-validation cohort | Internal held-out test; aggregate out-of-fold evaluation | 250 |
|  | Approximate outer-fold training sample | Training / development per outer fold | ~200 |
|  | Approximate outer-fold held-out test sample | Held-out test per outer fold | ~50 |
| **Nakamura (2026)** | | | |
|  | Development cohort (2 hospitals) | Training / development | 621 |
|  | Independent third-hospital cohort | External clinical test | 335 |

*n = number of echocardiographic examinations or patients as reported in each study.*

** EchoNet-Dynamic and CAMUS contributed to technical component validation only (segmentation, view classification, LVEF measurement) and not to clinical validation of diastolic dysfunction grading or filling pressure estimation.*

**Supplementary Table S6. Quantitative descriptors of methodological heterogeneity across included studies**

| **Study** | **ML model type** | **Echo input parameters / features** | **Primary validation cohort size*** | **Validation type** |
| --- | --- | --- | --- | --- |
| **Salem Omar (2018)** | Ensemble (RF, ANN, SVM) | 14 STE-derived features | 44 | Exploratory invasive substudy / internal CV |
| **Pandey (2021)** | DeepNN (tabular) | 9 guideline-derived variables | 84 | External invasive cohort |
| **Jiang (2022)** | SVM, DT, XGB, DNN | 8 tabular variables | 5,798 | Internal split |
| **Tromp (2022)** | End-to-end DL (CNN) | 6 automated parameters | 31,241 | External multicohort |
| **Gruca (2023)** | PLS regression | 25 strain timepoints | 194 | Internal hold-out |
| **Qu (2023)** | 1D ResNet (FFANet) | A4C morphology curves | Internal held-out test set from the 8:2 split | Internal split |
| **Chen (2023)** | CNN + SVM | 12 automated / derived features | 388 | External prospective |
| **Gottlieb (2024)** | Embedded vendor AI | 4 parameters | 209 | Prospective single-centre |
| **Park (2024)** | Automated pipeline + rule-based grading | 8 parameters | 200 | External prospective |
| **Xu (2024)** | LR, GB, RF | 12 (tabular + NT-proBNP) | **≈46 per iteration -** internal test set in each repeated split | Internal repeated splits |
| **Cai (2024)** | DL + RF (MMnet) | 6 automated multiview parameters | 239 | Internal test + external/public datasets |
| **Karuzas (2025)** | Commercial CNN | 8 parameters | 302 | Prospective single-centre |
| **Khan (2026)** | Eight supervised ML classifiers; logistic regression selected for detailed comparison | 47 candidate clinical, biomarker and echocardiographic features after prespecified filtering | 250 | Nested internal cross-validation; aggregate out-of-fold evaluation |
| **Nakamura (2026)** | XGBoost with SHAP explainability | Model 1: 5 parameters; Model 2: 9 parameters | 335 | Independent external multicentre test cohort |

Summary statistics: Number of input parameters/features: range 4–47, median 9. Primary validation cohort size: range 44–31,241, median 224. Predominant ML model types: end-to-end deep learning (n=4 studies); tabular ML or ensemble (n=6); hybrid DL and rule-based or ML (n=2); commercial or embedded AI (n=2).

** Primary validation cohort size = the primary validation or test set rather than total study sample including training data. For studies with multiple external cohorts, the largest single clinical validation cohort is reported. Number of echo input parameters reflects features entering the final discriminative model where reported.*

*Abbreviations: A4C = apical four-chamber view; ANN = artificial neural network; CNN = convolutional neural network; CV = cross-validation; DL = deep learning; DNN = dense neural network; DT = decision tree; FFANet = frequency-focused attention network; GB = gradient boosting; LR = logistic regression; ML = machine learning; MMnet = multi-task hybrid deep learning and machine learning model; NT-proBNP = N-terminal pro-B-type natriuretic peptide; PLS = partial least squares regression; RF = random forest; STE = speckle-tracking echocardiography; SVM = support vector machine; XGB = XGBoost.*

**Supplementary Material S7: PRISMA-DTA Checklist**

| **Section/topic** | **#** | **PRISMA-DTA Checklist Item** | **Reported on page #** |
| --- | --- | --- | --- |
| **TITLE / ABSTRACT** | | |  |
| Title | 1 | Identify the report as a systematic review (+/- meta-analysis) of diagnostic test accuracy (DTA) studies. | 1 |
| Abstract | 2 | Abstract: See PRISMA-DTA for abstracts. | 1-2 |
| **INTRODUCTION** | | |  |
| Rationale | 3 | Describe the rationale for the review in the context of what is already known. | 3 |
| Clinical role of index test | D1 | State the scientific and clinical background, including the intended use and clinical role of the index test, and if applicable, the rationale for minimally acceptable test accuracy (or minimum difference in accuracy for comparative design). | 3 |
| Objectives | 4 | Provide an explicit statement of question(s) being addressed in terms of participants, index test(s), and target condition(s). | 4 |
| **METHODS** | | |  |
| Protocol and registration | 5 | Indicate if a review protocol exists, if and where it can be accessed (e.g., Web address), and, if available, provide registration information including registration number. | 4 |
| Eligibility criteria | 6 | Specify study characteristics (participants, setting, index test(s), reference standard(s), target condition(s), and study design) and report characteristics (e.g., years considered, language, publication status) used as criteria for eligibility, giving rationale. | 4-5 |
| Information sources | 7 | Describe all information sources (e.g., databases with dates of coverage, contact with study authors to identify additional studies) in the search and date last searched. | 5 |
| Search | 8 | Present full search strategies for all electronic databases and other sources searched, including any limits used, such that they could be repeated. | Supplementary S1 |
| Study selection | 9 | State the process for selecting studies (i.e., screening, eligibility, included in systematic review, and, if applicable, included in the meta-analysis). | 5 |
| Data collection process | 10 | Describe method of data extraction from reports (e.g., piloted forms, independently, in duplicate) and any processes for obtaining and confirming data from investigators. | 5 |
| Definitions for data extraction | 11 | Provide definitions used in data extraction and classifications of target condition(s), index test(s), reference standard(s) and other characteristics (e.g. study design, clinical setting). | 4-6 |
| Risk of bias and applicability | 12 | Describe methods used for assessing risk of bias in individual studies and concerns regarding the applicability to the review question. | 5-6 |
| Diagnostic accuracy measures | 13 | State the principal diagnostic accuracy measure(s) reported (e.g. sensitivity, specificity) and state the unit of assessment (e.g. per-patient, per-lesion). | 5-6 |
| Synthesis of results | 14 | Describe methods of handling data, combining results of studies and describing variability between studies. This could include, but is not limited to: a) handling of multiple definitions of target condition. b) handling of multiple thresholds of test positivity, c) handling multiple index test readers, d) handling of indeterminate test results, e) grouping and comparing tests, f) handling of different reference standards | 6 |

Page 1 of 2

| **Section/topic** | **#** | **PRISMA-DTA Checklist Item** | **Reported on page #** |
| --- | --- | --- | --- |
| Meta-analysis | D2 | Report the statistical methods used for meta-analyses, if performed. | N/A |
| Additional analyses | 16 | Describe methods of additional analyses (e.g., sensitivity or subgroup analyses, meta-regression), if done, indicating which were pre-specified. | 6 |
| **RESULTS** | | |  |
| Study selection | 17 | Provide numbers of studies screened, assessed for eligibility, included in the review (and included in meta-analysis, if applicable) with reasons for exclusions at each stage, ideally with a flow diagram. | 7 |
| Study characteristics | 18 | For each included study provide citations and present key characteristics including: a) participant characteristics (presentation, prior testing), b) clinical setting, c) study design, d) target condition definition, e) index test, f) reference standard, g) sample size, h) funding sources | 7-11 |
| Risk of bias and applicability | 19 | Present evaluation of risk of bias and concerns regarding applicability for each study. | 18 |
| Results of individual studies | 20 | For each analysis in each study (e.g. unique combination of index test, reference standard, and positivity threshold) report 2x2 data (TP, FP, FN, TN) with estimates of diagnostic accuracy and confidence intervals, ideally with a forest or receiver operator characteristic (ROC) plot. | 12-17 |
| Synthesis of results | 21 | Describe test accuracy, including variability; if meta-analysis was done, include results and confidence intervals. | 15-17 |
| Additional analysis | 23 | Give results of additional analyses, if done (e.g., sensitivity or subgroup analyses, meta-regression; analysis of index test: failure rates, proportion of inconclusive results, adverse events). | 17-18 |
| **DISCUSSION** | | |  |
| Summary of evidence | 24 | Summarize the main findings including the strength of evidence. | 19-21 |
| Limitations | 25 | Discuss limitations from included studies (e.g. risk of bias and concerns regarding applicability) and from the review process (e.g. incomplete retrieval of identified research). | 21 |
| Conclusions | 26 | Provide a general interpretation of the results in the context of other evidence. Discuss implications for future research and clinical practice (e.g. the intended use and clinical role of the index test). | 21-22 |
| **FUNDING** | | |  |
| Funding | 27 | For the systematic review, describe the sources of funding and other support and the role of the funders. | 22 |

**Supplementary Table S8. Study-by-outcome matrix showing analytic-arm contributions to each evidence stratum.**

| **Study (Year)** | **Stratum 1 Invasive haemodynamic reference** | **Stratum 2 Guideline-label / surrogate** | **Stratum 3 Parameter-level replication** |
| --- | --- | --- | --- |
| **Pandey et al. (2021)** | **✓** | — | — |
| **Xu et al. (2024)** | **✓** | — | — |
| **Gruca et al. (2023)** | **✓** | — | — |
| **Khan et al. (2026)** | **✓** | — | — |
| **Nakamura et al. (2026)** | **✓** | — | — |
| **Salem Omar et al. (2018)** | — | **✓** *(surrogate label; invasive substudy reported descriptively only)* | — |
| **Jiang et al. (2022)** | — | **✓** | — |
| **Chen et al. (2023)** | — | **✓** | — |
| **Gottlieb et al. (2024)** | — | **✓** | — |
| **Karužas et al. (2025)** | — | **✓** | — |
| **Qu et al. (2023)** | — | **✓** | — |
| **Cai et al. (2024)** | — | **✓** *(final LVDF grading)* | **✓** *(parameter AUCs)* |
| **Park et al. (2024)** | — | **✓** *(LVDF grading)* | **✓** *(parameter AUCs)* |
| **Tromp et al. (2022)** | — | — | **✓** |

Individual studies may contribute to more than one evidence stratum because assignments were made at the analytic-arm level rather than the publication level. Reported AUC ranges in the main manuscript were derived from the primary diagnostic estimate within each stratum.

## Supplementary Table S9. Funding sources and relevant commercial involvement in included studies

| **Study** | **Funding/support reported** | **Commercial/vendor involvement or relevant conflicts reported** |
| --- | --- | --- |
| **Cai et al. 2024** | Beijing Key Specialists in Major Epidemic Prevention and Control; Hospital Science and Technology Innovation Fund. | Two authors were employees of **GE HealthCare China**. |
| **Chen et al. 2023** | Ministry of Industry and Information Technology of China; Ministry of Science and Technology of China; National Natural Science Foundation of China. | Several authors were full-time employees of **BioMind Technology**. |
| **Gottlieb et al. 2024** | No industry support sought. | Evaluated commercially available **Mindray Auto-EF/Auto-DFR** software; no author conflicts reported. |
| **Gruca et al. 2023** | None declared. | Author-level disclosures included Philips Medical Systems grant/speaker support and employment. |
| **Jiang et al. 2022** | Canadian Institutes of Health Research–Natural Sciences and Engineering Research Council Collaborative Health Research Project grant. | No conflicts of interest reported. |
| **Karužas et al. 2025** | European Union Horizon Europe programme; European Union/Lithuanian state budget funds. | Evaluated **Ligence Heart** software; several authors were employees of **Ligence Ltd**. |
| **Khan et al. 2026** | South-Eastern Norway Regional Health Authority grants. | Author-level disclosures included related patents and a GE Healthcare speaker honorarium. |
| **Nakamura et al. 2026** | Japan Society for the Promotion of Science KAKENHI grants. | Author-level disclosures included an endowed department, industry research grants, and honoraria outside the submitted work; no specific commercial developer of the model was reported. |
| **Pandey et al. 2021** | National Science Foundation; interinstitutional Smart Health Initiative; NHLBI Big-Data Challenge award; additional individual author grant support. | Author-level disclosures included Hitachi Healthcare grant support, consultancy, advisory-board roles, and research funding. |
| **Park et al. 2024** | Korea government/IITP grant; funders reported no role in the study. | Strong commercial involvement with **Ontact Health**, including employment/affiliation, patents, and stock ownership. |
| **Qu et al. 2023** | National Natural Science Foundation of China; Liaoning Province Applied Basic Research Program. | No known competing financial interests or personal relationships reported. |
| **Salem Omar et al. 2018** | Specific study funding not reported in available full text. | Author-level disclosures included consultancy, honoraria, equity interests, and industry research grants. |
| **Tromp et al. 2022** | A*STAR Biomedical Research Council and A*STAR Exploit Technologies; funder reported no role in study conduct or reporting. | Strong commercial involvement with **Us2.ai**, including employment, equity, patents, advisory roles, and logistical support. |
| **Xu et al. 2024** | None. | No conflicts of interest reported. |

**Abbreviations:** AI, artificial intelligence; COI, conflict of interest; EF, ejection fraction; IITP, Institute of Information & Communications Technology Planning & Evaluation; JSPS, Japan Society for the Promotion of Science; KAKENHI, Grants-in-Aid for Scientific Research; NHLBI, National Heart, Lung, and Blood Institute.

**Suggested table note:** Funding sources and commercial involvement are reported as disclosed in the original included publications. Commercial involvement includes author employment, equity, patents, consultancy, honoraria, grant support, or use of vendor-integrated/proprietary AI systems where reported. “Not reported” indicates that no specific study funding statement was identified in the available full-text article.
